# HCMV triggers frequent and persistent UL40-specific unconventional HLA-E-restricted CD8 T-cell responses with potential autologous and allogeneic peptide recognition

**DOI:** 10.1371/journal.ppat.1007041

**Published:** 2018-04-30

**Authors:** Nicolas Jouand, Céline Bressollette-Bodin, Nathalie Gérard, Magali Giral, Pierrick Guérif, Audrey Rodallec, Romain Oger, Tiphaine Parrot, Mathilde Allard, Anne Cesbron-Gautier, Nadine Gervois, Béatrice Charreau

**Affiliations:** 1 Centre de Recherche en Transplantation et Immunologie (CRTI), UMR1064, INSERM, Université de Nantes, Nantes, France; 2 Institut de Transplantation Urologie Néphrologie (ITUN), CHU Nantes, Nantes, France; 3 CRCINA, UMR1232, INSERM, Université d’Angers, Université de Nantes, Nantes, France; 4 LabEx Immunology-Graft-Oncology (IGO), Nantes, France; 5 Laboratoire de Virologie, CHU Nantes, Nantes, France; 6 Etablissement Français du Sang (EFS), Région des Pays de la Loire, Nantes, France; 7 Institut Hospitalo-Universitaire European Center for Science in Transplantation and Immunology, Nantes, France; Oregon Health Sciences University, UNITED STATES

## Abstract

Immune response against human cytomegalovirus (HCMV) includes a set of persistent cytotoxic NK and CD8 T cells devoted to eliminate infected cells and to prevent reactivation. CD8 T cells against HCMV antigens (pp65, IE1) presented by HLA class-I molecules are well characterized and they associate with efficient virus control. HLA-E-restricted CD8 T cells targeting HCMV UL40 signal peptides (HLA-E_UL40_) have recently emerged as a non-conventional T-cell response also observed in some hosts. The occurrence, specificity and features of HLA-E_UL40_ CD8 T-cell responses remain mostly unknown. Here, we detected and quantified these responses in blood samples from healthy blood donors (n = 25) and kidney transplant recipients (n = 121) and we investigated the biological determinants involved in their occurrence. Longitudinal and phenotype *ex vivo* analyses were performed in comparison to HLA-A*02/pp65-specific CD8 T cells. Using a set of 11 HLA-E/UL40 peptide tetramers we demonstrated the presence of HLA-E_UL40_ CD8 αβT cells in up to 32% of seropositive HCMV^+^ hosts that may represent up to 38% of total circulating CD8 T-cells at a time point suggesting a strong expansion post-infection. Host’s HLA-A*02 allele, HLA-E *01:01/*01:*03* genotype and sequence of the UL40 peptide from the infecting strain are major factors affecting the incidence of HLA-E_UL40_ CD8 T cells. These cells are effector memory CD8 (CD45RA^high^RO^low^, CCR7^-^, CD27^-^, CD28^-^) characterized by a low level of PD-1 expression. HLA-E_UL40_ responses appear early post-infection and display a broad, unbiased, Vβ repertoire. Although induced in HCMV strain-dependent, UL40_15-23_-specific manner, HLA-E_UL40_ CD8 T cells are reactive toward a broader set of nonapeptides varying in 1–3 residues including most HLA-I signal peptides. Thus, HCMV induces strong and life-long lasting HLA-E_UL40_ CD8 T cells with potential allogeneic or/and autologous reactivity that take place selectively in at least a third of infections according to virus strain and host HLA concordance.

## Author summary

Understanding the mechanisms of immune control of viral infection is crucial to improve diagnosis and to design efficient immunotherapies. CD8 T lymphocytes are key components of the cellular immunity against human cytomegalovirus (HCMV), a widespread pathogen that cause severe illness and poor outcome in immunocompromised hosts such as transplant recipients and HIV-infected patients. In this study we characterized a population of non-conventional CD8 T lymphocytes directed against the viral protein UL40 and presented by the non-classical HLA-E molecules in blood samples from HCMV seropositive hosts. This immune response was detectable in around 30% of hosts, may represent up to 38% of total blood CD8 T lymphocytes, persists for life and thus seems to belong to the common immune arsenal against HCMV. Genetic factors related to the host and to the different strains of HCMV are critical parameters for the existence of this immune subset. Although specifically induced in response to HCMV infection, a key feature of these cells is their potential ability to be also responsive against multiple HLA molecules. In conclusion, HCMV infection frequently leads to the long-term persistence of a large subset of lymphocytes with potential side effect requiring attention in contexts such as autoimmunity and transplantation.

## Introduction

Human cytomegalovirus (HCMV; human herpesvirus 5) is the prototype member of β-herpesvirus and a widespread opportunistic pathogen. In healthy individuals, primary infection is asymptomatic and is followed by a life-long, persistent, infection that is controlled by host immune system [1]. Nevertheless, HCMV is a major cause of morbidity and mortality in immunocompromised individuals such as transplant recipients or HIV-infected patients. HCMV is the most common cause of congenital infection in the world that can result in neurodevelopmental delay and sensorineural hearing loss. HCMV disease can manifest in many forms, including infectious mononucleosis, hepatitis, post-transplant arteriosclerosis, pneumonia, colitis, immune senescence, and alteration to the immune repertoire [1]. The impact of HCMV on the outcome of solid organ transplantation (SOT) is substantial. HCMV not only causes a highly morbid and potentially fatal illness but also indirectly influences other relevant outcomes, such as allograft acute and chronic rejection, other opportunistic infections, post-transplant lymphoproliferative disorders, vascular disease, and overall patient and allograft survival [2, 3]. Because of the magnitude of its direct and indirect impacts, there have been extraordinary efforts to define strategies for its prevention, monitoring and treatment [1, 4].

Cellular immune response is the major mechanism by which HCMV replication is controlled [5, 6]. Large human HCMV-specific T-cell responses have been described in numerous published reports, particularly in the transplantation setting and in ageing [7, 8]. HCMV-specific T cells in healthy adults can constitute as much as 10% of the total memory CD4 and CD8 T cells that recognize multiple viral proteins, notably, pp65, IE1, IE2 and gB [9, 10]. Suppression of the number and function of HCMV-specific CD4 and CD8 T cells allows reactivation of the virus from latency, leading to uncontrolled viral replication and clinical disease in immunocompromised hosts, including SOT recipients [5].

The CD8 T-cell response appears as the most important component of the anti-HCMV immune response [7], although CD4 T cells and natural killer (NK) cells also play a significant role [11, 12]. Expanded HCMV-specific responses are often thought to be a requirement for protection and could result from the life-long latency of HCMV in specific cells, interspaced with episodic reactivations that gradually increase response size in a process called inflation [13, 14]. HCMV-specific T cells were first described as those able to recognize the immunodominant antigen immediate early 1 (IE1) [15], but later studies emphasized the importance of T cells that target a tegument phosphoprotein of 65kDa (pp65/UL83) [16]. The original epitope identification studies focused on NLVPMVATV, a HLA-A*02 restricted epitope within pp65 that was defined as a “typical” epitope because of its common detection in HLA-A*02–positive individuals. The identification of other less common epitopes targeted by HCMV-specific T cells has extended the panel of HCMV-reactive T cells in humans [9, 10]. In murine models, subdominant epitopes have been shown to be protective [17]. Despite technical advances in terms of HCMV-specific T-cell response monitoring [18], a correlation between T-cell responses and clinical protection has not been established to date. This underlines a need for a global analysis of anti-HCMV T cell responses at both qualitative and quantitative levels investigating response numbers, sizes, hierarchy levels, peptide specificities, time course and duration.

HCMV-specific CD8 T cells directed against UL40 epitopes presented by HLA-E have more recently emerged as an additive piece in the complexity of anti-HCMV immune response [19]. HLA-E is a poorly polymorphic non-classical (MHC-Ib) HLA molecule. Although more than 20 *HLA-E* alleles have been registered, only two nonsynonymous *HLA-E* alleles: HLA-E*01:01 (HLA-E^107R^) and HLA-E*01:03 (HLA-E^107G^) that differ by a single amino acid (R107G) have been found in most populations [20]. Cell surface expression of HLA-E depends on binding of a conserved 9-mer peptide naturally provided by the N-terminal signal peptide of classical HLA-I or HLA-G molecules. HCMV UL40 signal peptide contains a 9-mer sequence with an exact sequence identity to endogenous HLA-E–binding peptides. The prototype of UL40 peptide loaded on HLA-E molecules is VMAPRTLIL provided by the AD169 HCMV strain [21]. As a consequence, HCMV UL40 promotes efficient cell surface expression and stabilization of HLA-E independently of TAP function [21, 22]. HLA-E containing peptides engage two types of receptors. HLA-E binds the NK cell inhibitory receptor CD94/NKG2A [23, 24] and, thereby, promotes efficient protection against lysis by CD94/NKG2A^+^ NK cells [22, 23, 25, 26]. In addition to CD94/NKG2A, HLA-E interacts with CD94/NKG2C, albeit with lower affinity. CD94/NKG2C is an activating receptor predominantly expressed on a relatively small population of NK cells. Interestingly, the frequency of this CD94/NKG2C^+^ NK subset increases in HCMV-infected individuals [27] [6]. HLA-E/UL40 (HLA-E_UL40_) complexes also trigger TCR-dependent activation of a subset of CD8 αβ T cells [28–30]. UL40-specific/anti-HCMV HLA-E–restricted CD8 cytotoxic T-cell responses have been reported in healthy donors and in kidney and lung transplant recipients and associated with a possible harmful impact on graft endothelial cells [30] and allograft survival [31]. Characterization of these CD8 T-cell subsets in healthy and transplanted population remains sparse and no longitudinal study is available. In healthy hosts, the beginning and duration of HCMV infection are usually unknown, thus monitoring the development of T-cell responses starting at the time of infection is not possible except in the setting of organ transplantation where post-graft primary HCMV infections are frequent and require a specific follow-up.

Our study investigated the presence of circulating HLA-E-restricted CD8 T cells in a cohort of kidney transplant recipients (KTR, n = 121) during either an active HCMV infection (at primary infection or at reactivation) or at latency and in HCMV seropositive (HCMV^+^) healthy volunteers (HV, n = 25). Using a set of HLA-E tetramers refolded with 11 different UL40 epitopes to cover the diversity of HCMV clinical strains, we provide here a quantitative analysis of HLA-E_UL40_-restricted anti-HCMV T-cell response in hosts. The frequency, the magnitude, the time course of HLA-E_UL40_-restricted anti-HCMV CD8 T-cell responses, as well as the phenotype and the specificity of peptide recognition of these subsets, were documented *ex vivo* in comparison to the conventional HLA-A*02_pp65_ CD8 T-cell responses. Altogether our findings reveal that HCMV induces early long-lasting HLA-E–restricted, UL40-specific unconventional CD8 T-cell responses that often parallels HLA-I-restricted CD8 T cells. Although their induction seems initially restricted by both the viral infecting strain and host’s HLA-I, extended peptide recognition may occur allowing these effector responses to potentially target self and allogeneic, donor-specific, HLA-I peptides.

## Results

### HCMV infection induces frequent and long lasting HLA-E_UL40_-restricted T-cell responses

**Fig 1:**
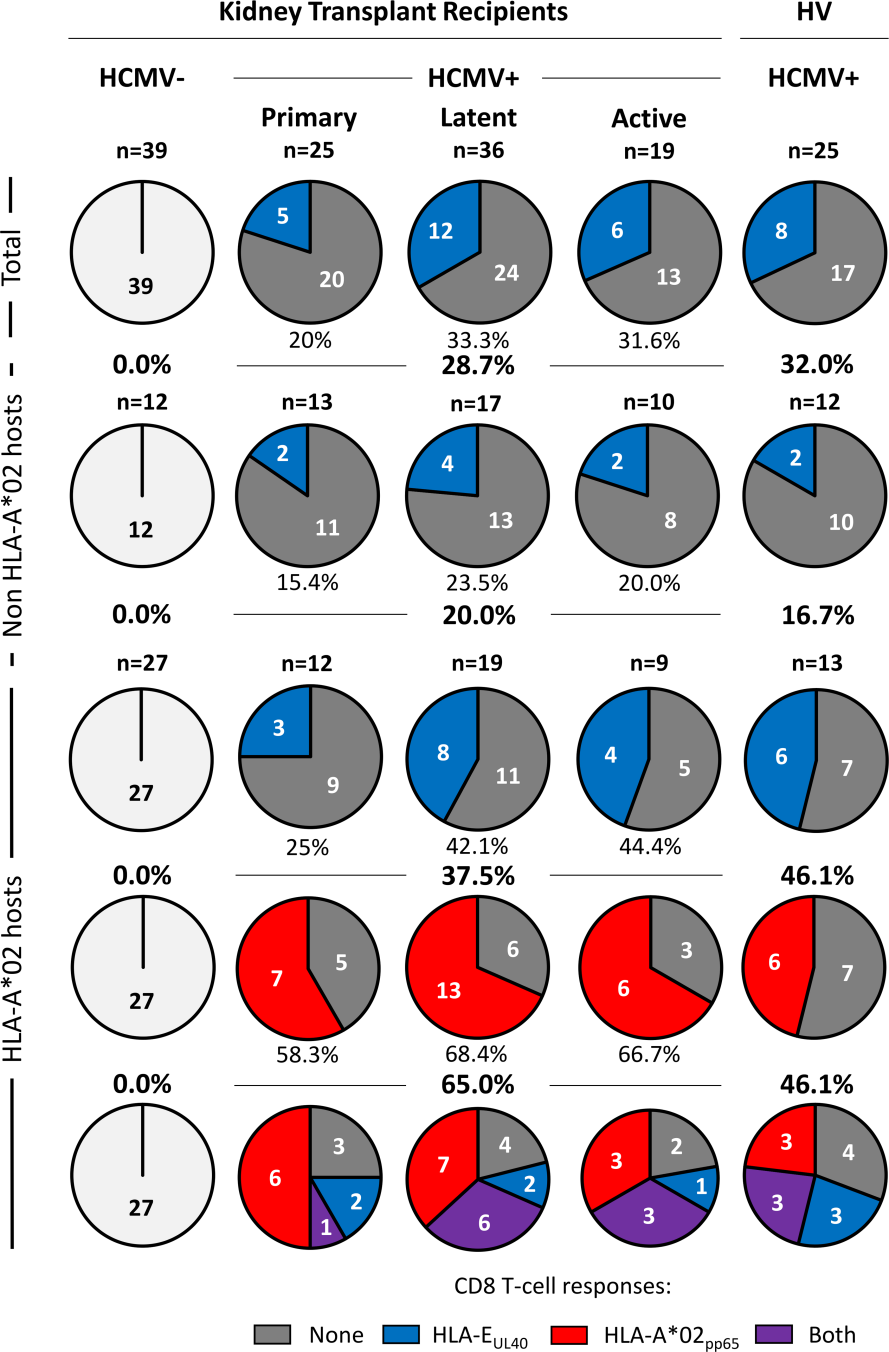
Frequency of unconventional HLA-E_UL40_ CD8 T-cell responses compared to conventional HLA-A*02_pp65_ CD8 T-cell responses in HCMV^+^ kidney transplant recipients and healthy volunteers. PBMCs were isolated from freshly or prospectively harvested at M12 post-transplantation blood samples issued from healthy donors (HV) or from kidney transplant recipients (KTR), respectively. *Ex vivo* detection of HLA-E_UL40_ CD8 T and HLA-A*02_pp65_ CD8 T cells was performed using flow cytometry by selecting CD3^+^ CD8α^+^ TCRγδ^-^ tetramer^+^ cells on PBMCs. Detection threshold was 0.1% of total CD8 αβT cells and kidney transplant recipients and healthy volunteers bearing ≥0.1% of HLA-E_UL40_ CD8 T cells (in blue) or ≥0.1% HLA-A*02_pp65_ CD8 T cells (in red) were considered as positive. Detection of both types of CD8 T-cell responses are indicated in violet. Absence of detection is shown in light grey in HCMV^-^ recipients and dark grey for HCMV^+^ hosts. Data shown are the number of individuals that display anti-HCMV CD8 T-cell responses. Frequencies of the CD8 T-cell subsets were calculated among subgroups for all (total), non HLA-A*02 and HLA-A*02 individuals and expressed as percentages (%).

Although UL40-specific HLA-E-restricted CD8 T-cells have been described in a few HCMV seropositive (HCMV^+^) individuals [28–30, 32], only sparse data are available concerning their characteristics and post-infection occurrence. To address this point, we performed a retrospective detection and quantification of circulating HLA-E_UL40_-restricted CD8 T-cell responses in a cohort of kidney transplant recipients (KTR, n = 121) and in HCMV^+^ healthy volunteers (HV, n = 25). Our study cohort included transplanted patients segregated into 4 groups according to recipient’s HCMV serology (HCMV^-^ and HCMV^+^) and, for HCMV^+^ patients, the status of infection (primary, latent, active) at 12 months post-transplantation. Demographic and clinical characteristics of the cohort are presented in **Table 1**. UL40-specific HLA-E-restricted CD8 T cells were analysed *ex vivo* in blood samples after PBMC isolation using a multi-parameter (CD3^+^CD8α^+^TCRγδ^-^) flow cytometry assay subsequent to the blockade of the CD94 receptor using a specific blocking mAb. Our protocol was adapted from Pietra *et al*. [29] and allows a sensitive (threshold of detection: 0.1% of total CD8 TCRαβ T cells) and peptide-specific analysis of HLA-E_UL40_ CD8 T-cell populations (**S1 Fig**). Detection of HLA-A*02:01/pp65 CD8 T (HLA-A*02_pp65_) cells was carried out in parallel for a comprehensive analysis of unconventional (HLA-E-restricted) *versus* conventional (HLA-A*02:01-restricted) anti-HCMV responses. Banked blood samples, harvested at M12 post-transplantation, were investigated using a set of HLA-E tetramers loaded with 8 different UL40_15-23_ peptides to encompass the usual UL40_15-23_ variability among common HCMV strains [33, 34]. The 8 HLA-E tetramer/peptide complexes were tested individually. **Fig 1** shows the distribution of HLA-E_UL40_ CD8 T-cell responses (detected for at least 1 tetramer HLA-E/UL40 peptide complex) *versus* HLA-A*02_pp65_ CD8 T-cell responses in the various groups. In HCMV^-^ transplanted patients no HLA-E_UL40_ nor HLA-A*02_pp65_ T-cell response was detected, consistent with the concept that these responses are induced by and specific to HCMV infection. HLA-E_UL40_ CD8 T cells were detectable in all HCMV^+^ subgroups (primary, latency, reactivation) and were present in an overall of 28.7% of HCMV^+^ transplanted recipients and 32.0% in HCMV^+^ blood donors. By comparison the overall incidence of HLA-A*02_pp65_ T-cell responses in HLA-A*02 patients and healthy hosts was 65.0% and 46.1%, respectively. HLA-A*02_pp65_ T-cell responses were roughly similar in frequency upon primary (58.3%), latent (68.4%) and active (66.7%) infection while HLA-E_UL40_ CD8 T-cell responses were lower upon primary infection compared to other groups. Interestingly, HLA-E_UL40_ CD8 T-cell responses was more frequent in HLA-A*02 as compared to non HLA-A*02 hosts (37.5% *versus* 20.0% for transplant recipients and 46.1% *versus* 16.7% for HV; p = 0.0318). In HLA-A*02 recipients, HLA-E_UL40_ CD8 T-cell responses were found either associated with (32.2%) or independent (16.1%) of a HLA-A*02_pp65_ T-cell response. Coexistence of HLA-E_UL40_ and HLA-A*02_pp65_ CD8 T-cell responses also occurs in 33.3% of healthy hosts. Together these results reveal a very high incidence (up to 46%) of HLA-E_UL40_ CD8 T-cell responses in HCMV^+^ hosts with no significant difference between transplanted patients and healthy individuals suggesting that antiviral and immunosuppressive regimens have no impact of these cell subsets at M12. These cells are detected more frequently in hosts carrying an HLA-A*02 allele. Unconventional CD8 T cells can be detected independently of detectable conventional HLA-A*02_pp65_ T-cell response. Presence of HLA-E_UL40_ CD8 T cells early post-infection (primary or reactivation) as well as at latency suggests long lived cell subsets consistent with memory anti-HCMV response. Of interest, the lack of HLA-E_UL40_ CD8 T cells in HCMV^-^ transplant recipients may also indicate that, although a full sequence homology between common UL40_15-23_ peptides and signal peptides from most HLA-A, -B and -C molecules, presentation of allogeneic (i.e. donor) HLA-I signal peptides (HLAsp) by HLA-E-expressing uninfected cells in the graft doesn’t drive the generation of consequent anti-donor HLA-E_HLAsp_ CD8 T-cell response.

**Table 1:** Patient’s characteristics.

			HCMV^+^		
	HCMV^-^ n = 39	Primary-infection * n = 27	Absence of HCMV infection n = 36	Reinfection/ Reactivation * n = 19	p-value
*H**CMV serologic status (M12 post transplantation)*					
D-/R-; ***R-***	28	/	/	/	
D+/R-; ***R-***	11	/	/	/	
D-R- or D-/R+; ***R+***	/	1	16	10	
D+/R- or D+/R+; ***R+***	/	26	20	9	
*D**onors*					
Age [years; median (Q1-Q3)]	44.8 (36.3–55.5)	56.7 (45.6–64.6)	49.7 (41.0–58.5)	57.7 (44.0–64.5)	**0.0102**^**1**^
Gender [Male/Female; (% of Male)]	26/13 (66.7%)	12/15 (44.4%)	18/18 (50.0%)	11/8 (57.9%)	0.2874^2^
Donor status [Deceased/Living; (% of deceased donors)]	39/0 (100.0%)	26/1 (96.3%)	36/0 (100.0%)	18/1 (94.7%)	0.1426^2^
*R**ecipients*					
Age [years; median (Q1-Q3)]	45.0 (35.4–54.8)	49.2 (43.3–66.8)	56.0 (40.9–63.2)	57.2 (45.7–61.9)	0.0960^**1**^
Gender [Male/Female; (% of Male)]	24/15 (61.5%)	21/6 (77.8%)	24/12 (66.7%)	10/9 (52.6%)	0.3275^2^
Transplant [Kidney/Pancreas-Kidney; (% of Kidney only)]	29/10 (74.3%)	27/0 (100.0%)	33/3 (91.7%)	16/3 (84.2%)	**0.0100**^2^
Cold ischemia [minutes; median (Q1-Q3)]	1086 (813–1463)	1068 (852–1412)	1140 (901–1503)	974 (869–1256)	0.6536^**1**^
Serum Creatinine at M12 [μmol/L; median (Q1-Q3)]	121 (105–140)	137 (121–170)	136 (106–171)	152 (121–175)	0.1395^**1**^
Proteinuria at M12 [g/24h; median (Q1-Q3)]	0.14 (0.11–0.26)	0.28 (0.12–0.47)	0.19 (0.11–0.40)	0.26 (0.16–0.48)	0.1190^**1**^
**Immunosuppressive regimen [n;(%)]**					
Unknown	6 (15.4%)	19 (70.4%)	6 (16.7%)	18 (94.7%)	**< 0.0001**^**3**^
None	2 (5.1%)	0 (0.0%)	1 (2.8%)	0 (0.0%)	
Basiliximab/Simulect	18 (46.1%)	6 (22.2%)	19 (52.8%)	0 (0.0%)	
(ATG)/Thymoglobulin	13 (33.3%)	2 (7.4%)	10 (27.8%)	1 (5.3%)	
HLA-A*02 recipients¶ [n;(%)]	27 (69.2%)	12 (44.4%)	19 (52.8%)	9 (47.4%)	0.1743^2^
Total HLA-I mismatches [n; median (Q1-Q3)]	3 (2–3.5)	3 (2.5–4)	3 (2–3)	3 (2.5–4)	0.0652^**1**^
Total HLA-II mismatches [n; median (Q1-Q3)]	3 (3–4)	3 (2–3)	3 (2–3)	3 (1–3)	0.1381^**1**^
Donor Specific Antibodies (DSA) [n;(%)]	0 (0.0%)	1 (3.7%)	1 (2.8%)	4 (21.1%)	**0.0079**^2^
Post-Tx HCMV infection [n;(%)]	0 (0.0%)	27 (100.0%)	/	/	**< 0.0001**^2^
HCMV infection time post-Tx [months; median (Q1-Q3)]	/	7 (3–9)	/	7 (5–10)	0.8536^4^
Post-Tx HCMV reactivation [n;(% of the HCMV^+^ recipients)]	/	22 (81.5%)	/	19 (100%)	0.0674^2^
**HCMV anti-viral prophylaxis [n;(%)]**					
None	27 (69.2%)	3 (11.1%)	2 (5.5%)	0 (0.0%)	**<0.0001**^**3**^
Ganciclovir	0 (0.0%)	1 (3.7%)	0 (0.0%)	2 (10.5%)	
Valganciclovir/Rovalcyte	12 (30.8%)	23 (85.2%)	34 (94.4%)	17 (89.5%)	

D: Donor; R: Recipient

^1^ Kruskall-Wallis test

^2^ Fisher’s exact test

^3^ Pearson’s chi-squared test

^4^ Mann Whitney test

¶ transplant recipients carrying at least one HLA-A*02 allele

*Definitions of Cytomegalovirus infection [35]

### HLA-A*02 and HLA-E genotypes are major determinants associated with HLA-E_UL40_ CD8 T-cell responses

**Fig 2:**
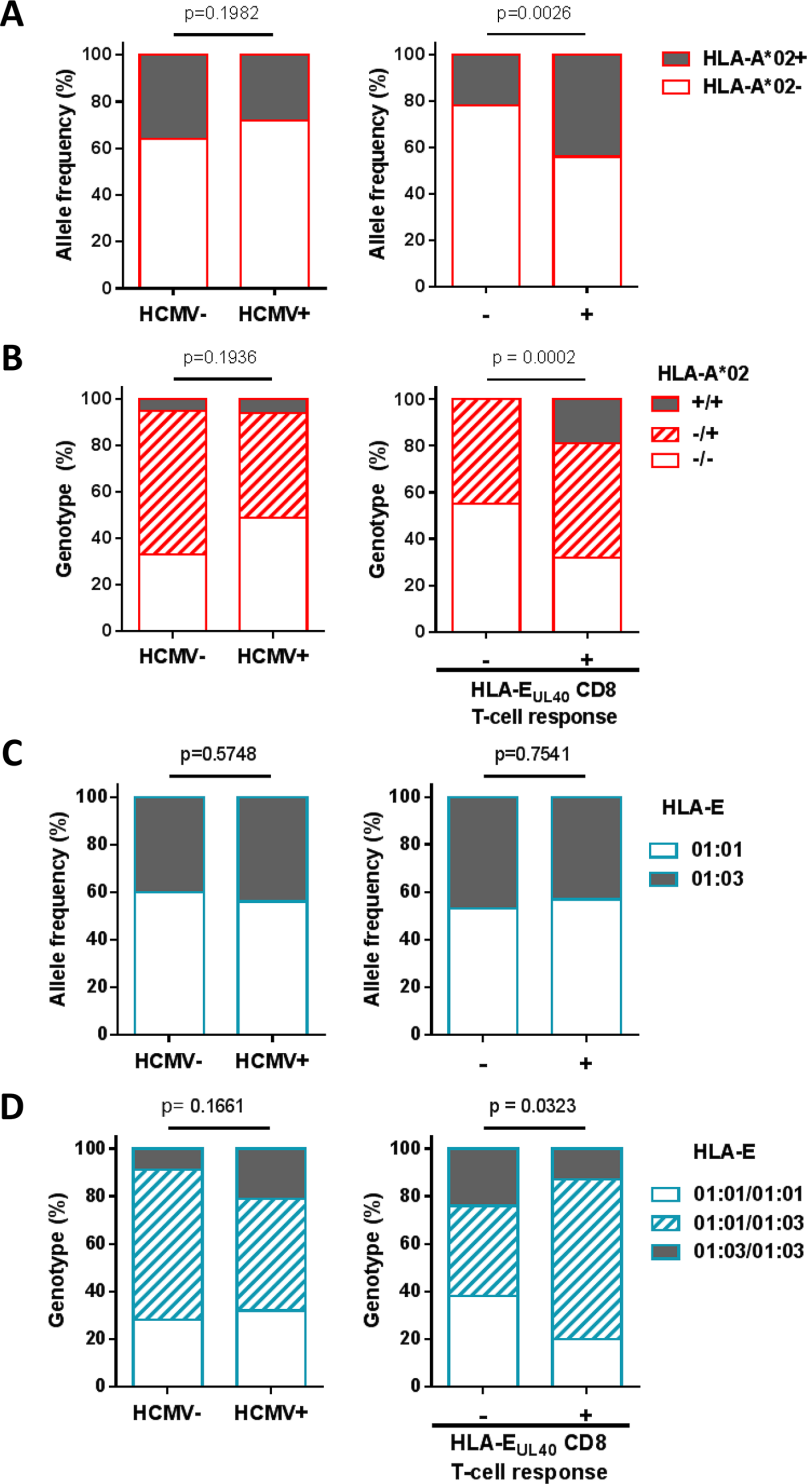
HLA-A*02 allele and HLA-E genotype influence the development of HLA-E_UL40_-specific CD8 T cells in HCMV^+^ individuals. (A) HLA-A*02 allele frequency and (B) genotype distribution were investigated in HCMV^-^ (n = 39) *versus* HCMV^+^ (n = 105) individuals including a total of 144 healthy volunteers and kidney transplant recipients (left panels) and in HCMV^+^ host with (+, n = 31) or without (-, n = 74) HLA-E_UL40_ CD8 T-cell response (right panels). (C) HLA-E*01:01 and HLA-E*01:03 allele frequency and (D) HLA-E genotype distribution were investigated in HCMV^-^ (n = 35) *versus* HCMV^+^ (n = 96) individuals of the cohort (left panels) and in HCMV^+^ host with (+, n = 30) or without (-, n = 66) HLA-E_UL40_ CD8 T-cell response (right panels). *P* values were calculated using appropriate statistical tests: Fisher’s exact test for allele frequencies and Chi-square test for genotype distribution analysis.

Detection of HLA-E_UL40_ CD8 T cells suggested that these unconventional responses occur more frequently in HLA-A*02 carriers. Genotyping of HLA-A was then performed to decipher this association. Firstly, HLA-A*02 allele frequency was 28% in the HCMV^+^ hosts in our study, a value similar to those found in the HCMV^-^ counterpart (36%, p = 0.1982) (**Fig 2A**). Our findings indicate that HLA-A*02 allele frequency was significantly higher in HCMV^+^ hosts with HLA-E_UL40_ responses than in non-responders (44% versus 22%, *p* = 0.0026, **Fig 2A**). Next, distribution of HLA-A*02 genotypes were compared between HCMV^+^ and HCMV^-^ individuals. A similar distribution of HLA-A*02 genotypes was observed in both groups **(Fig 2B**). However, HCMV^+^ hosts that display HLA-E_UL40_ responses were more frequently hosts carrying two HLA-A*02 alleles than hosts without response (19% *versus* 0%, p = 0.0002). Similar analysis was performed for HLA-E alleles and genotypes. HLA-E sequencing allowed us to discriminate the two major HLA-E*01:01 and HLA-E*01:03 alleles. These variants differ in a single amino acid at position 107 when an arginine (R) in HLA-E*01:01 is replaced by a glycine (G) in HLA-E*01:03 resulting in different thermal stabilities and lengths of interaction with cognate receptors [33]. HLA-E allele distribution was found equal for HCMV^-^ and HCMV^+^ hosts (**Fig 2C,** left panel) and no difference in HLA-E allele frequency was observed among HCMV^+^ individuals with or without HLA-E_UL40_ responses (57.0% *versus* 53.0%, and 43.0% *versus* 47.0%, for HLA-E*01:01 and *01:03 respectively, p = 0.7541, **Fig 2C,** right panel). An equal distribution of HLA-E genotypes was calculated for HCMV^+^ and HCMV^-^ hosts (p = 0.1661) (**Fig 2D,** left panel). However, a significant change occurs in HLA-E genotypes for hosts that display or not HLA-E_UL40_ responses (p = 0.0323) with a higher prevalence of heterozygous HLA-E*01:01/HLA-E*01:03 in hosts with HLA-E_UL40_ responses (**Fig 2D, right panel**). No impact of donor HLA was found. These findings support a role for immunogenetic factors in the occurrence of HLA-E_UL40_ responses upon HCMV infection and associate HLA-A*02/A*02 and HLA-E*01:01/HLA-E*01:03 genotypes as independent *(p* = 0.85) positive factors promoting HLA-E_UL40_ responses.

### HLA-E_UL40_ CD8 T cells target the highly polymorphic UL40_15-23_ sequence in a strain-specific manner

**Fig 3:**
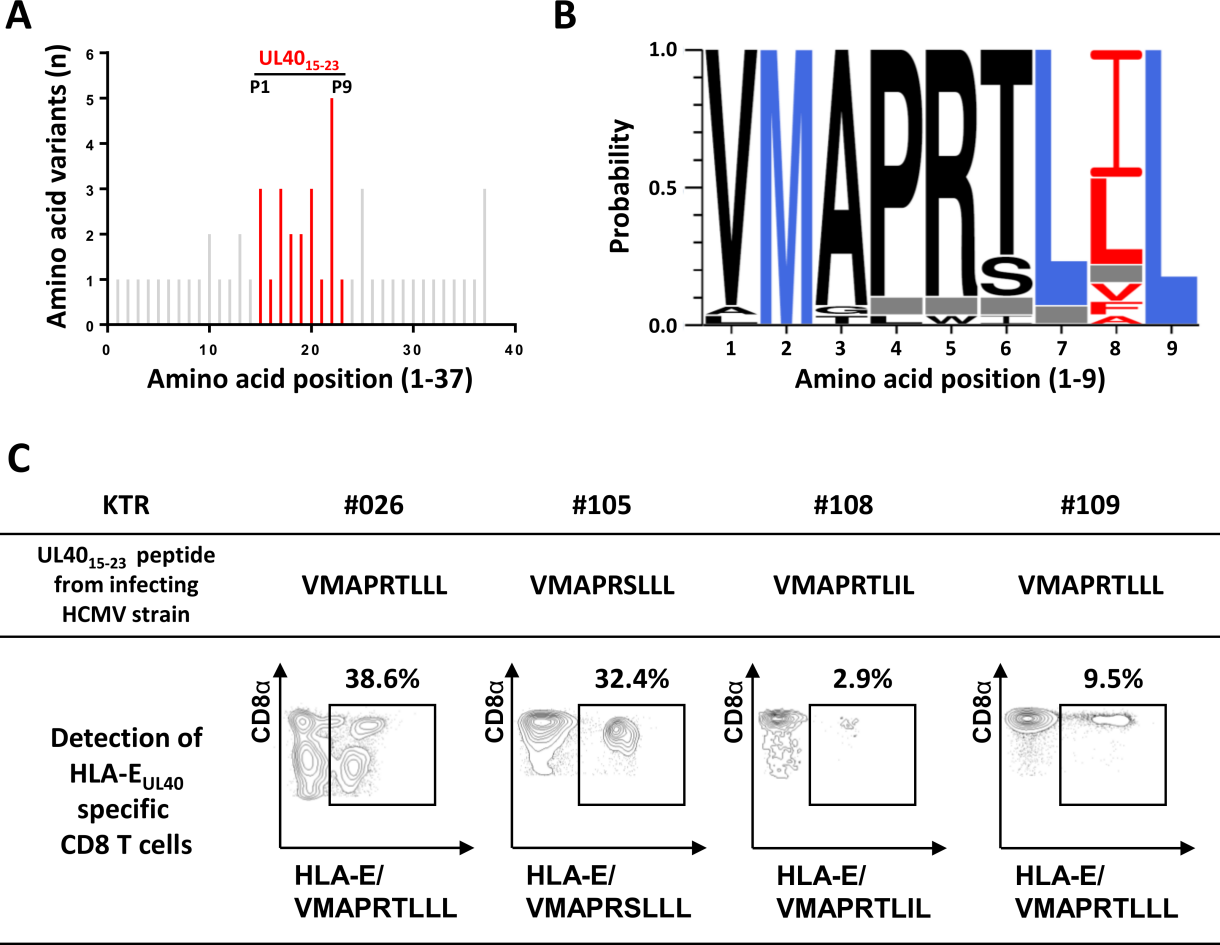
HCMV strain-dependent variability of UL40_15-23_ sequences and HCMV strain-specific HLA-E_UL40_ T-cell response in hosts. (A, B) Genomic DNAs isolated from HCMV positive blood samples in HCMV^+^ transplant recipients (n = 25) were sequenced for the identification of UL40 protein (amino acids 1–221) provided by the circulating HCMV strains. (A) Amino acid variability, expressed as a number of amino acid variants, within the HLA-E-binding peptide (UL40_15-23_, shown in red) among the sequence for HCMV UL40 signal peptide (UL40_1-37_, shown in grey). A total of 32 UL40 sequences from 25 hosts were analysed. UL40 protein sequence from the Merlin HCMV clinical strain was used as reference. Positions 1 to 9 of residues in the HLA-E-binding peptide (UL40_15-23_) are indicated. (B) Sequence LOGO of the UL40_15-23_ HLA-E-binding peptide from 25 transplanted hosts. The height of the letter is proportional to the frequency of each amino acid in a given position (P1 to P9). Major anchor residues for binding in the HLA-E peptide groove are indicated in blue. Red letters highlight the important variability observed in position 8 of the HLA-E-binding peptide. Grey boxes correspond to a constitutive deletion of the corresponding amino acid in the UL40 sequence from the infecting viral strain. (C) Representative dot plot analyses showing the detection of strain-specific anti-UL40 HLA-E-restricted CD8 T-cell responses in 4 KTRs (KTR#026, #105, #108 and #109). Frequencies (%) of the HLA-E_UL40_-specific T cells among total circulating αβ CD8 T cells are indicated.

Next, we sought to determine the specificity of HLA-E_UL40_ CD8 T-cell responses toward UL40 peptide provided by the host’s HCMV infecting strain. To this aim, DNAs isolated from whole blood samples from transplant recipients undergoing either a primary infection (n = 18) of a reactivation (n = 7) of HCMV during the 12 months post-transplantation were used for UL40 protein (AA 1–221) sequencing. Sequencing identified a total 32 UL40 sequences for the 25 infected patients, some patients carrying more than one infecting strain (**Table 2**). Overall variability of full UL40 protein among strains is reported in **S2 Fig** and was consistent with a previous report [34]. UL40 variability affects 38 AA along the sequence but mostly concentrates within the region encoding the signal peptide (UL40_1-37_) and in particular inside UL40_15-23,_ the HLA-E binding epitope (**Fig 3A**). Notably, AA22 and to a lesser extend AA20 that correspond to the peptide position P8 and P6, respectively, two critical residues for the interaction with the CD94/NKG2-A or -C or with the TCR of specific T cells [32] [36], were the most variable, with respectively 48.2% and 19.6% of AA variability and up to 5 and 3 different AA (**Fig 3A and 3B**). In contrast, residues 16 (P2), 21 (P7) and 23 (P9) that correspond to the 3 major anchor AA for the peptide binding pockets of HLA-E, were highly conserved [37]. Three major UL40_15-23_ sequences (VMAPRTLIL, VMAPRTLLL, VMAPRSLLL) accounted for 62.5% of the HCMV strains detected in patients (15 out of 25) while 10 other UL40 sequences were found only in a single patient (**Table 2**). These data confirmed that consensus UL40_15-23_ sequences such as VMAPRTLIL and VMAPRTLLL are highly predominant in clinical strains. Interestingly, VMAPRTLIL and VMAPRTLLL UL40 sequences are fully homologous to signal peptide sequence for the majority of HLA-A and HLA-C alleles excluding the most common HLA-A*02 and HLA-C*07. Since banked blood samples were available for 23 of these patients, we next assessed the presence of HLA-E_UL40_ CD8 T-cell responses using dedicated HLA-E/UL40 tetramers. HLA-E_UL40_ CD8 T cells were detected in 6 out of the 23 patients (26.1%) and illustrated for 4 out of the 6 in the **Fig 3C**. As shown in **Fig 3C**, when HLA-E_UL40_ CD8 T-cell responses were investigated using HLA-E tetramers loaded with the UL40 peptide that we identified in their own infecting strain, HCMV strain-specific HLA-E-restricted T cells were detected in patients. Importantly, percentages of HLA-E_UL40_ CD8 T cells vary from 2.9% up to 38.6% of total CD8 αβT cells in the blood sample at the time of detection. Fig 3C also illustrates the complexity of the patterns of HLA-E_UL40_ CD8 T responses. Indeed, while in a large majority of hosts, homogenous CD8α bright populations were observed exemplified in patients #108 and #109, in few hosts, such as #026, multiple populations that display various levels of CD8α expression (low and high) were observed. This may reflect either the detection of concomitant subsets of HLA-E_UL40_ CD8 T at a particular time point or different stage of activation for a single population or both. Thus, our data sustain previous report showing the UL40_15-23_ nonapeptide as a highly polymorphic region inside the viral UL40 protein [34]. Our data show that UL40 polymorphism also drives (strain-specific) antigen-specific HLA-E-restricted T cells. However, in our study only a limited set of canonical UL40 peptides were found in the majority of clinical infecting strains (such as VMAPRTLIL, VMAPRTLLL and VMAPRSLLL identified in 20 out of 32 strains) and allowed strain-specific HLA-E_UL40_ CD8 T cells. Interestingly, about a third of patients were infected by an HCMV strain carrying a non-canonical UL40_15-23_ sequence that display variant amino acid on the residues P1, P3, P4, P5, P6 and P8. Thus we speculate that such HCMV strains for which no HLA-E_UL40_ CD8 T-cell response was detected in our assays may hold UL40_SP_ probably not able to bind HLA-E. Nevertheless, we cannot rule out the possibility that detection of HLA-E_UL40_ CD8 T cells was underestimated in our study due to the lack of tetramers loaded with the full set of UL40 sequences identified in clinical isolates.

**Table 2:** Sequences of UL40_15-23_ peptide in the infecting HCMV strains.

UL40_15-23_ signal Peptide*	HCMV strains n/32	HLA-E_UL40_ CD8 T-cell responders n/6
VMAPRTLIL	9	1
VMAPRTL**L**L	7	2
VMAPR**S**L**L**L	4	1
VMA**——**L	2	0
VMAPR**I**LIL	1	1
VMAPRTL**A**L	1	1
VMAPRTL**F**L	1	0
VMAPRTL**V**L	1	0
VM**G**PRTLIL	1	0
VMA**L**RTLIL	1	0
VM**T**PRTL**V**L	1	0
VMAP**WS**LIL	1	0
**A**MAPRTLIL	1	0
**L**MAPRTL**F**L	1	0

*Variant amino acids compared to canonical VMAPRTLIL UL40_15-23_ sequence are shown in bold.

### Time course and magnitude of HLA-E_UL40_ and conventional HLA-A*02_pp65_ CD8 T-cell responses are parallel

**Fig 4:**
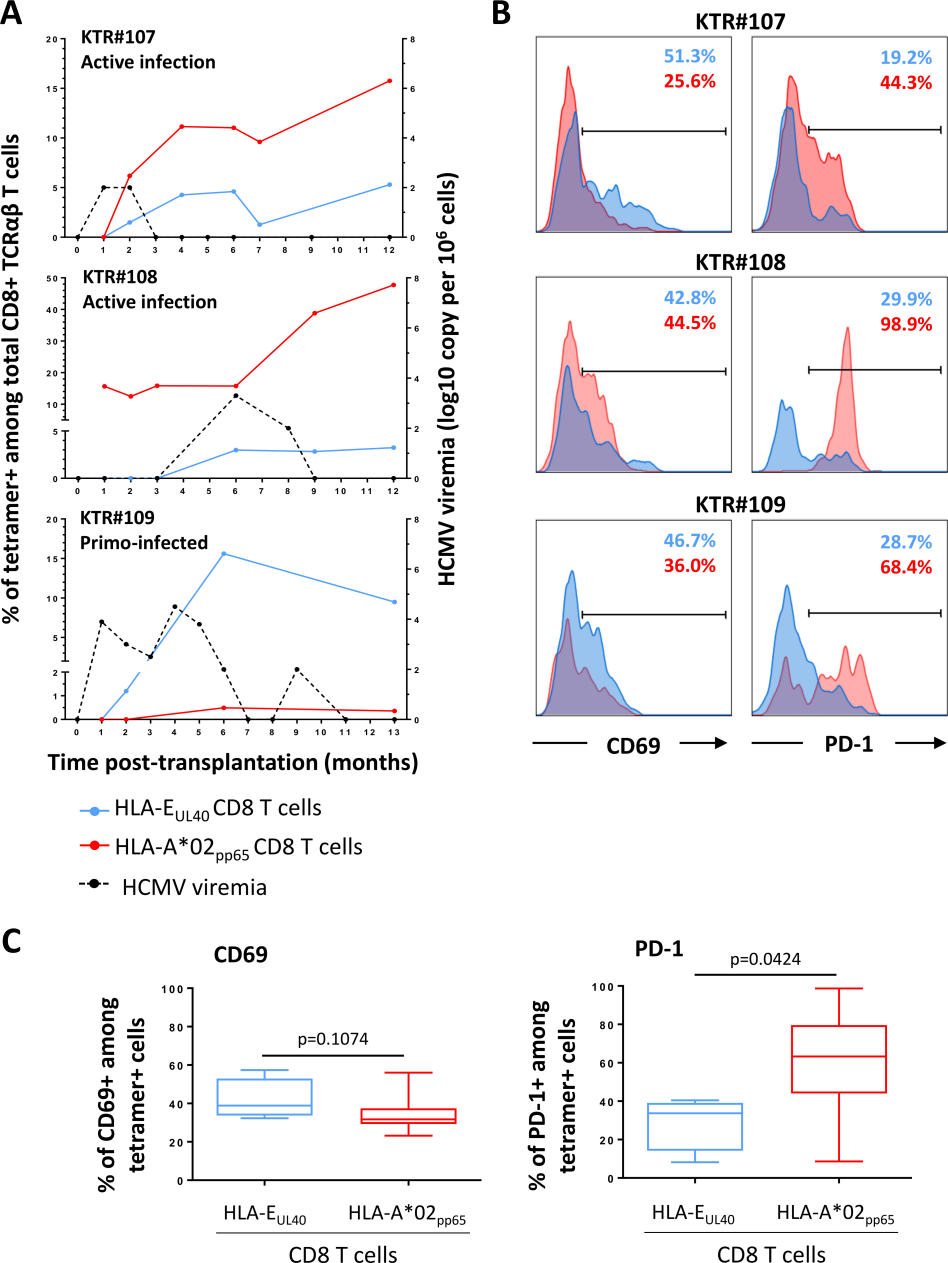
Time course analysis of the HLA-E_UL40_ and HLA-A*02_pp65_ CD8 T-cell anti-HCMV responses upon infection and patterns of activation markers. (A) Time course analysis of the HLA-E_UL40_ and HLA-A*02_pp65_ CD8 T-cell responses according to the HCMV viremia. PBMCs prospectively collected from M0 and M13 (#109) post-transplantation were retrospectively processed for the concomitant detection and quantification of anti-HCMV HLA-E_UL40_ and HLA-A*02_pp65_ CD8 T-cell responses upon infection. Three representative patterns of anti-HCMV CD8 T cell responses in 3 KTR (KTR#107, #108 and #109) are represented. (B) Analysis of T-cell activation. Expression of CD69 (left panel) and PD-1 (right panel) analysed on blood samples from KTR#107, #108 and #109. Facs histogram overlays represent the % of expression for the activation markers CD69 and PD-1 among CD3^+^ CD8α^+^ TCRγδ^-^ tetramers^+^ cells, for HLA-E_UL40_ (in blue) and HLA-A*02_pp65_ (in red) anti-HCMV CD8 T-cell responses at M6 post-transplantation. (C) Comparative analysis of CD69 (left panel) and PD-1 (right panel) expression on HLA-E_UL40_ (n = 4 hosts) and HLA-A*02_pp65_ (n = 8 hosts) CD8 T cells investigated at M6 post-transplantation. *P* values were calculated using a Mann Whitney test.

To further characterize the HLA-E-restricted anti-HCMV T-cell responses, time course of these responses during the acute phase of infection and beyond, and T-cell activation markers were monitored post-infection in patients (n = 16) with either a primate infection or a reactivation of the virus. Results from 3 patients are illustrated in the **Fig 4A** that summarizes the most frequent profiles that we observed. Upon primary infection (exemplified by patient #109), HLA-E_UL40_ CD8 T cells develop early and most often concomitantly to HLA-A*02_pp65_ T-cell response. HLA-E_UL40_ CD8 T cells are detected in blood 1 month post-infection (patient #107 and #109) and may even precede detection of HLA-A*02_pp65_ T-cell response (patient #109). HLA-E_UL40_ CD8 T-cell response can be either predominant (patient #109) or lower in frequency among total CD8 αβT cells compared to conventional HLA-A*02_pp65_ response (patients #107 and #108). Patient #108 illustrates a HCMV reactivation with a pre-existing HLA-A*02_pp65_ population leading to a clear increase in the percentage of HLA-A*02_pp65_ CD8 T cells at the time of reactivation and a *de novo* induction of HLA-E_UL40_ CD8 T cells. For the 3 patients, consistent long term (M9-12 post-infection) responses were observed ranging from 1.2 to 15.6% for HLA-E_UL40_ CD8 T cells and 0.4 to 47.7% HLA-A*02_pp65_ CD8 T cells. Activation markers (CD69 and PD-1) were analysed by flow cytometry for both anti-HCMV CD8 T-cell subsets at each time point. **Fig 4B** reports on the relative expression of CD69 and PD-1 investigated *ex vivo* at M6 post-transplantation for the 3 recipients. Overall, we found that both subsets display similar rate of CD69^+^ cells. In contrast, there were striking differences in the programmed death-1 (PD-1) expression between the 2 subsets with a lower percentage of expression for PD-1 on HLA-E_UL40_ CD8 T cells as compared to HLA-A*02_pp65_ CD8 T cells (**Fig 4C**). These discrepancies were found at all time points post-induction (**S3 Fig**). More than 45% of HLA-A*02_pp65_ CD8 T-cell subsets express sustained levels of PD-1^+^ after a primary infection (patients #107 and #109) and up to 100% upon reactivation (patient #108). These investigations that shape the temporal occurrence of HLA-E_UL40_ CD8 T cells post-infection reveal that both responses, conventional and unconventional, may be very close in kinetic, persistence and in percentage of total CD8 T cells in blood. However, although similarly activated early post-infection, low expression of PD-1 could be a feature of HLA-E restricted anti-HCMV T-cell responses.

### UL40_15-23_-specific HLA-E-restricted CD8 T cells are effector memory T cells with broad TCR Vβ repertoire and peptide recognition

The functional and phenotype description of HLA-E_UL40_ CD8 T cells is rather limited. Our phenotypic analyses by flow cytometry, performed *ex vivo* for 3 patients (#107, #108 and #109) confirmed that HLA-E_UL40_ T cells belong to the CD3^+^CD4^-^CD8αβ^+^TCRαβ^+^ T cell subset. HLA-E_UL40_ T cells exhibited a phenotype (CR45RA^high^CD45RO^low^CD27^-^CD28^-^CD57^+/-^CCR7^-^, **S4 Fig**) consistent with effector memory T-cell response as previously reported [38]. Furthermore, in our study, to better characterize anti-HCMV HLA-E-restricted responses, HLA-E_UL40_ CD8 T-cell lines were generated by cell sorting using for each patient an HLA-E tetramer loaded with the UL40 peptide identified in their own HCMV circulating strain. (**Fig 3C**). PBMCs from 5 HCMV^+^ patients with a primary infection or a reactivation (KTR #104, #105, #107, #108, #109) were sorted and then amplified *in vitro* to reach a purity>95% (defined by tetramer staining using the HLA-E/UL40 complexes employed for sorting).

**Fig 5:**
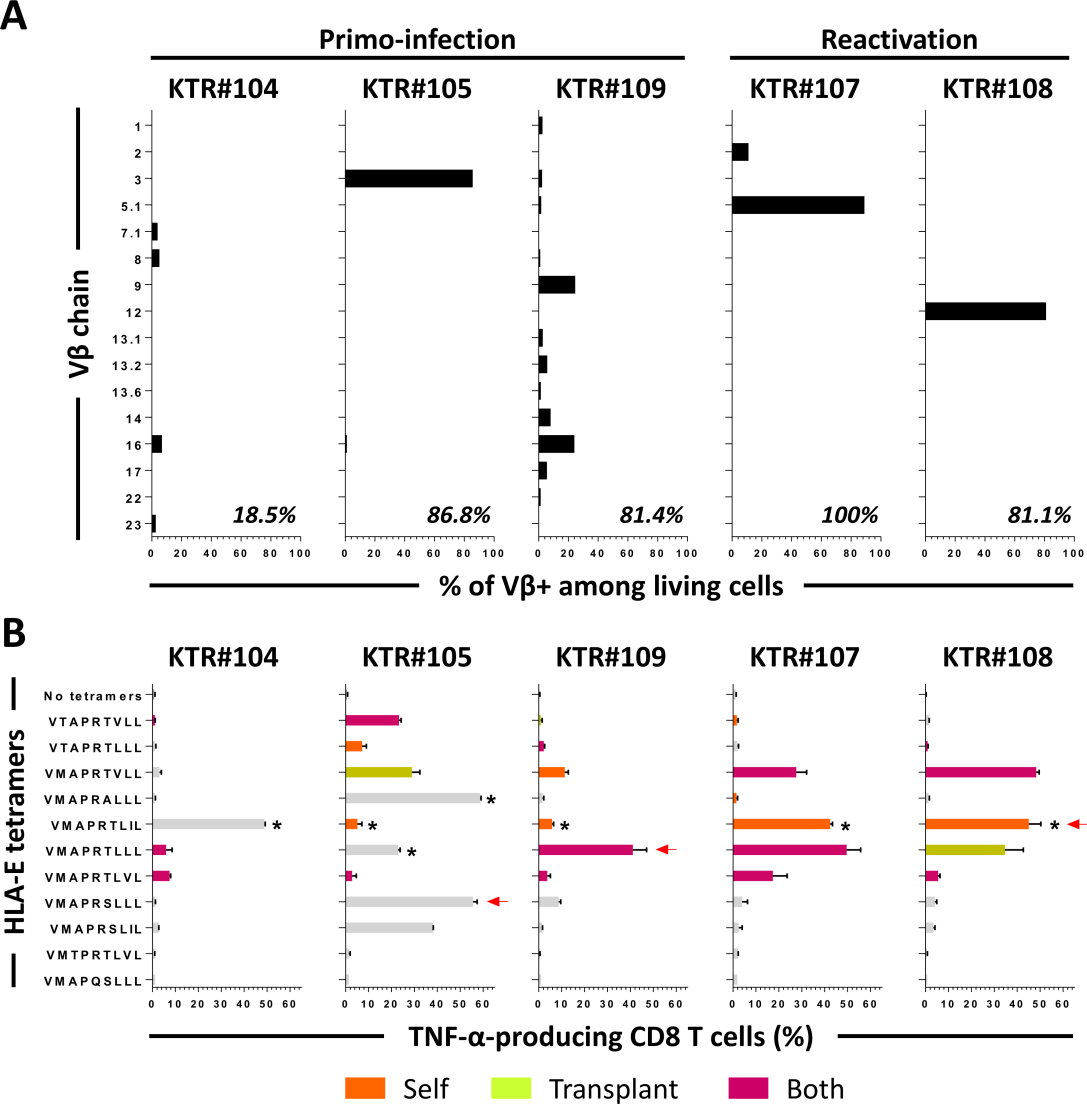
Diversity of HLA-E_UL40_ CD8 TCR Vβ repertoire and peptide recognition. HLA-E_UL40_ CD8 T cells were sorted from PBMCs for 5 KTR (#104, #105, #107, #108 and #109) and amplified *in vitro*. (A) Diversity of the HLA-E_UL40_-specific TCR Vβ repertoire. Percentages indicate the ratio of individual Vβ chains by the total repertoire for each patient. (B) Specificity of peptide recognition toward UL40 and HLA-I peptides. HLA-E_UL40_-specific CD8 T cells were stimulated for 5h with one of the eleven HLA-E_UL40_ tetramers and the percentage of TNF-producing cells among total CD8α cells was determined. Red arrows indicate the HCMV UL40 peptide provided by the infecting strain. Recognition of HLA-Ia signal peptides derived from autologous (orange bars), transplant-specific allogeneic (green bars) or both (purple bars) is shown. Grey bars indicate recognition of peptides that do not match with UL40 from the infecting strain or with HLA-Ia signal peptides not expressed by donors or hosts. Asterisks indicate samples with missing data for HLA-C genotype.

Amplified HLA-E_UL40_ T cells were used for the analysis of T-cell receptor β chain variable region (TCR-Vβ) expression by flow cytometry using 24 antibodies reactive to 70% of the human TCR-Vβ repertoire. Given the fact that *HLA-E* is a poorly polymorphic gene and that HLA-E_UL40_ CD8 T cells recognize a restricted number of UL40_15-23_ peptides, the question of the existence of a public T-cell repertoire between individuals was raised. Consistently, only few analysis of TCR sequences from UL40-specific T-cell clones have been reported yet and display a limited number of TCR including Vβ3, 5.1, 9, 16, 22 [29]. HLA-E_UL40_ CD8 T-cell population expressing a dominant Vβ chain sub-family was obtained for 3 patients while another one gives rise to oligoclonal populations (from 2 to 6 subsets detected) with variable distribution (**Fig 5A**). This suggests the sorting of multiple, coexisting, HLA-E_UL40_ CD8 T-cell populations in this patient. Interestingly, a broad TCR-Vβ repertoire was found with 16 Vβ identified (Vβ1, 2, 3, 5.1, 7.1, 8, 9, 12, 13.1, 13.2, 13.6, 14, 16, 17, 22 and 23) thus enlarging the Vβ repertoire previously described for these cells. For patient #104 that exhibits oligoclonal T-cell populations only 19% of Vβ repertoire was identified suggesting that this patient carry a dominant Vβ not detectable in our assay. No dominant Vβ was identified for patient #109 with oligoclonal HLA-E-restricted subsets covering 82% of its repertoire.

Next, amplified HLA-E_UL40_ CD8 T-cell populations were investigated for their capacity to produce TNF in response to TCR engagement in a peptide-specific manner. To this aim, the 5 enriched populations were stimulated with 11 HLA-E/UL40 peptide tetramers, used individually, for 5h before intracellular TNF staining. The set-up of experimental conditions are depicted in **S5 Fig**. In most cases, T cells were highly stimulated (up to 50% of cells expressing TNF) by the HLA-E tetramers loaded with the peptide corresponding to UL40_15-23_ identified in their own infecting stain (**Fig 5B**). However, consistent stimulations (10 to 50%) were also obtained for HLA-E tetramers loaded with other peptides. Interestingly, T-cell activation can be induced by peptides that correspond to self and donor-specific allogeneic HLA_SP_ supporting the idea that these T cells may be auto- and/or alloreactive (**Fig 5B**). In most of cases, changing in P8 or P6 residues of UL40_15-23_ peptides diminished or abolished the reactivity of T cells, showing the relative importance of these two amino acids for the interaction of the HLA-E complexes with the TCR. Such cross-stimulation was observed similarly for T-cell populations containing a single dominant Vβ subset or oligoclonal subsets. Magnitude of the stimulation was peptide-dependent and differs for each T-cell subpopulation. In most cases the dominant peptide issued from the infecting strain and used for sorting, gives the highest score of T-cell activation. Together, these data may suggest that a single dominant Vβ subset of HLA-E_UL40_ CD8 T cells induced in a UL40 peptide-dependent manner could be activated by HLA-E molecules presenting UL40 peptides with a degree of homology including a panel of HLA_SP_.

### HLA-E_UL40_ CD8 T-cell responses display potential recognition of self and allogeneic HLA peptides

**Fig 6:**
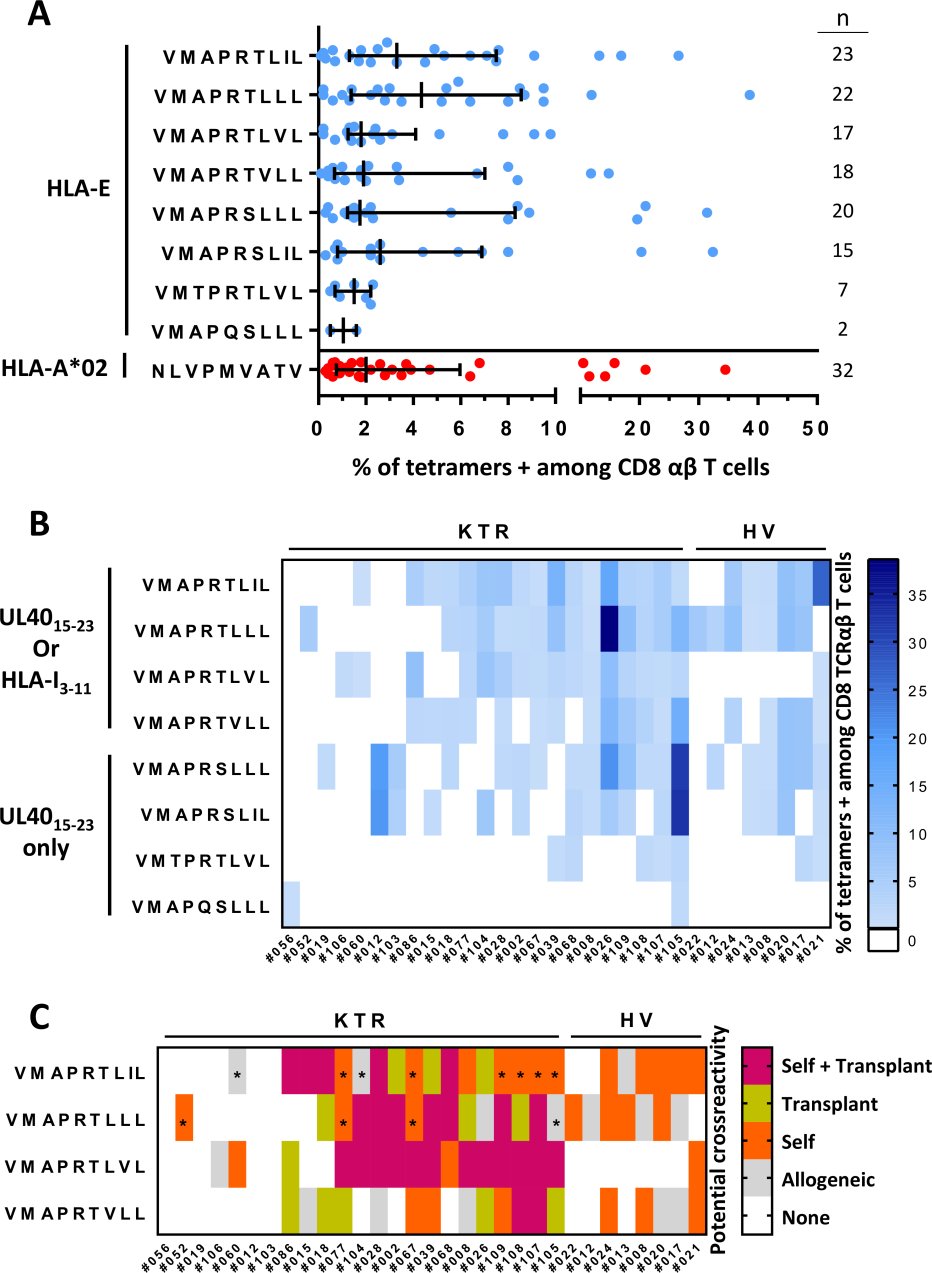
Potential cross-recognition of autologous and allogeneic HLA-I signal peptides by HLA-E_UL40_ CD8 T cells. PBMCs were isolated from freshly or prospectively collected blood samples at M12 post-transplantation issued from healthy donors (HV, n = 25) or from kidney transplant recipients (KTR, n = 119), respectively. *Ex vivo* detection of HLA-E_UL40_ CD8 T and HLA-A*02_pp65_-specific CD8 T cells was performed using flow cytometry by selecting CD3^+^ CD8α^+^ TCRγδ^-^ tetramer^+^ cells on PBMCs. Eight different HLA-E_UL40_ tetramers were used independently. (A) Percentage of circulating anti-HCMV CD8 T cells in blood detected using the various HLA-E_UL40_ (blue) and HLA-A*02_pp65_ (in red) tetramers in HV and KTR. For each tetramer/peptide, the number of individuals with a given CD8 T-cell response is indicated. (B) Diversity and magnitude of the HLA-E_UL40_ CD8 T-cell responses in KTR and HV. HLA-E_UL40_ CD8 T-cell responses appear in blue and colour intensity is proportional to the percentage of HLA-E_UL40_ CD8 T cells. (C) Classification of the HLA-E_UL40_ CD8 T-cell responses in HCMV^+^ hosts according to possible recognition of self (orange), donor-specific allogeneic (green) or both (violet) (n = 31, 23 KTR and 8 HV). Grey boxes show HLA-I signal peptides which are not derived from the recipient, nor from the donor. Asterisks indicate peptides with underestimated information due to a lack of HLA-C genotyping.

The use of 8 different HLA-E/UL40 peptide tetramers allowed us to decipher the spectrum of HLA-E_UL40_ responses generated post HCMV infection. This assay provided a qualitative and quantitative analysis of HLA-E-restricted responses for the 31 HCMV^+^ transplanted patients and HV initially found to carry at least one HLA-E_UL40_ CD8 T-cell response. Responses were analyzed to define, for each individual, both peptide specificity and relative strength of the responses (percentage of subset among total circulating CD8 T cells). As a result, consistent responses were observed for the 8 tetramers tested. VMAPRTLLL, VMAPRTLIL, VMAPRTLVL, VMAPRTVLL, VMAPRSLLL and VMAPRSLIL are the most frequently recognized peptides by HLA-E_UL40_ responses in terms of both occurrence and magnitude. The number of circulating HLA-E-restricted CD8 T cells varies in the range of 0.1% (detection threshold) up to around 40% of total TCRαβ CD8 T cells. These percentages were similar or even higher than those we obtained for HLA-A*02:01-restricted responses (**Fig 6A**).

An overview of the panel of HLA-E_UL40_ T-cell responses detected in patients and HV is provided in the **Fig 6B**. This analysis shows that HCMV^+^ subjects usually display HLA-E_UL40_ T-cell responses against more than a single HLA-E/peptide complex. The total number of responses (from 1 to 8) detected as well as the nature of UL40 peptide recognition (specificity and magnitude) is variable among the hosts. Similar variability is observed for HV and transplant recipient populations. These *ex vivo* findings sustained our results above obtained with cell lines and showing that a monoclonal HLA-E_UL40_ CD8 T-cell subset can be activated by a set of UL40 peptides. Nevertheless, we cannot exclude that a multiplicity of responses can also coexist in hosts resulting from coinfection.

Considering the ability of HCMV to generate HLA-E_UL40_ T-cell responses that recognize multiple peptides we next sought to determine whether the detected HLA-E_UL40_ CD8 T cells may target autologous or allogeneic (i.e. provided by the transplant donor) HLA-I signal peptide in the KTR. To this aim, sequence of HLA-I (-A, -B, -C) signal peptide carried by the host (KTR or HV) or by the transplant donors were compared to the UL40 sequences targeted by HLA-E_UL40_ CD8 T-cell responses to identify self and allogeneic, donor-specific or non donor-specific, peptides, respectively. Potential self or allogeneic recognition mediated by anti-HCMV HLA-E-restricted T-cell subset are presented in the **Fig 6C**. Due to full sequence homology between UL40 viral peptides and HLA-I signal peptides, most of UL40-induced responses were found to recognize at least one autologous HLA peptide for all HCMV^+^ individuals. Moreover, in most cases (70% of responders) HLA-E_UL40_ responses may also potentially target transplant HLA-I signal peptide presented by HLA-E molecules on the graft.

## Discussion

*Ex vivo* HLA-E_UL40_ tetramer staining allowed us to provide a qualitative and quantitative assessment of unconventional CD8 T cells directed against HCMV. This unconventional T-cell subset is restricted by the MHC-Ib, HLA-E molecule, and targets UL40 signal peptide (UL40_15-23_). A number of conclusions can be drawn from this study. First, a major finding was the high prevalence of this CD8 T-cell population investigated in HCMV^+^ transplant recipients and healthy volunteers. HLA-E_UL40_ CD8 T cells were detected in 31 out of 105 (29.5%) HCMV^+^ hosts. About half (46.1%) of HCMV^+^ healthy HLA-A*02 blood donors possesses detectable HLA-E_UL40_ CD8 T cells. An equal proportion of HLA-A*02 blood donors (46.1%) possess HLA-A*02_pp65_ CD8 T cells and 1/3 of these individuals display both anti-HCMV CD8 T cells. Although, in our cohort of transplant recipients, HLA-A*02_pp65_ specific T cells were more frequently detected than HLA-E_UL40_ CD8 T cells, the latter were found in over 35% of kidney transplant recipients. Nevertheless, we cannot exclude that these values were underestimated since ideally, a broader panel of HLA-E_UL40_ complexes would be used for an exhaustive detection. Moreover, T-cell populations below 0.1% (our threshold of detection) were not considered. Together these results support the idea that HLA-E-restricted T-cell response belongs to the usual T-cell response against HCMV UL40.

Conventional T-cell responses to HCMV peptides, such as dominant responses to the pp65 and IE epitopes presented by HLA-A*02 and HLA-B*07, can regularly reach 5–10% of total CD8 T cells in the blood of healthy adults and even greater with up to 30% of total CD8 T cells are reported in some studies [9, 10, 39]. However, there is extensive variability in the size of T-cell responses between individuals. The reasons for this variability are not fully understood but may include the dose and timing of infection, as well as the HLA restriction element. Here we show that similar disparity also occurs for HLA-E-restricted anti-UL40 CD8 T cells with frequencies varying from 0.1% to over 38% of total CD8 T cells according to the hosts (median value: 2.2%). These values are the highest reported for this unconventional subset *ex vivo*. Previous studies established *ex vivo* percentages of HLA-E-restricted anti-UL40 CD8 T cells from 0.05% [31] to 14% [30, 32]. Thus, HLA-E-restricted responses mirror HLA-Ia-restricted responses in both frequency and magnitude. Our longitudinal analysis demonstrated that these T-cell populations develop early post-infection and expand quickly to reach maximal rate between 2 to 11 months post primary infection and within 1 month post reactivation. Tetramer staining of HLA-E_UL40_ CD8 T cells showed continued expansion post-infection and stabilization at high frequencies. In our cohort study, anti-HCMV HLA-E-restricted, and to a lesser extent HLA-A*02-restricted T-cell responses appear more frequent during latent and reactivations/secondary infections than during primary infections. Although this difference may be due to variations in the time interval between infection and the time point selected for detection assay (M12) among individuals or an effect of immunosuppressive regimen, this could also reflect a delay in HLA-E_UL40_ CD8 T-cell induction.

A key point of this study is to provide evidence for a positive correlation between HLA-A*02 allele and the occurrence of HLA-E-restricted anti-HCMV CD8 T cells. Firstly, using HLA-E_UL40_ tetramer staining, anti-HCMV HLA-E-restricted were detected more often in HLA-A*02 hosts. Next, HLA sequencing further confirmed a significantly higher rate of hosts carrying at least one HLA-A*02 allele among HLA-E_UL40_ CD8 T-cell responders compared to non-responders. Moreover, all HLA-A*02^+/+^ HCMV^+^ individuals (n = 6) developed an HLA-E_UL40_ CD8 T-cell response. The positive correlation between HLA-A*02 allele and HLA-E_UL40_ CD8 T-cell response could be related to the sequence of HLA-A*02 signal peptide (VMAPRTLVL). Indeed, HLA-E_UL40_ CD8 T-cell responses that have been identified in HCMV infection typically involved epitopes that are structurally related to canonical HLA-I leader sequences but foreign to the hosts [19, 40, 41]. Consistent with the paucity of the VMAPRTLVL sequence among viral strains, UL40 sequencing of host’s circulating strains allowed us to identify the VMAPRTLVL sequence only in a single clinical strain out of 32. Thus, it could be suspected that the presence of HLA-A*02 decreases the chances that a host will present a signal peptide derived from a different HLA-I allele, one that could cause negative selection of HLA-E_UL40_ reactive TCR. In that respect, when HLA-A*02 is present, deletion of HLA-E_UL40_-responsive T cells is less likely.

HLA-E*01:01 (HLA-E^107R^) and HLA-E*01:03 (HLA-E^107G^) alleles only differ in a single amino acid at position 107 and the frequencies of these two variants are equal in most populations [20]. It has been shown that the HLA-E*01:03 variant is usually expressed at higher levels than HLA-E*01:01 [33]. Although located outside the peptide-binding groove, the mutant AA at position 107 may also possibly affect the conformation of HLA-E or its association with β2-microglobulin resulting in the presentation of different peptide repertoires [42]. We found no HLA-E allele preference associated with the establishment of an HLA-E_UL40_ CD8 T-cell response. Instead, we report a higher prevalence of HLA-E*01:01/*01:03 heterozygous among individuals carrying an HLA-E_UL40_ CD8 T-cell response. Interestingly, it has been demonstrated for HLA-E and for the non-human primate HLA-E ortholog that a large panel of identified peptides can be presented by all allotypes [43]. Both alleles present a limited set of peptides derived from class I leader sequences physiologically [42]. In stress conditions (viral infections, tumors), HLA-E can present peptides from other sources than the signal sequences of classical HLA-I molecules [38, 44]. Recent studies demonstrated that the HLA-E alternative peptide repertoire observed in pathophysiological conditions seems not to be shared equally by the two HLA-E alleles [42, 45]. Comparing the HLA-E*01:03-restricted peptides to those of HLA-E*01:01, Celik et al. demonstrated that the peptide repertoire of both alleles greatly differs in the absence of HLA class I molecules leading to functional disparity between both alleles [45]. Consistent with these observations, it is likely that bearing both *01:01/*01:03 alleles may improve HLA-E stability and the diversity of peptide presentation and thus increase HLA-E_UL40_ T-cell responses as suggested by our findings. In transplant recipients, the impact of donor HLA was investigated in parallel to the impact of recipient of donor HLA. We found no significant impact neither for HLA-A,-B,-C or HLA-E alleles nor for a mismatch between donor and recipient for HLA-A,-B,-C or HLA-E.

An elegant study from Wang *et al*. suggested that HCMV-specific CD8 TCR repertoire diversity is more important than CD8 T-cell response magnitude for the control of persistent HCMV infection [46]. Using a single-cell based approach for the clonotype analysis of human CD8 TCRαβ repertoires they demonstrate a high prevalence of both TCRα and TCRβ public motif usage. Our analysis of TCR Vβ usage by HLA-E_UL40_-specific T cells investigated after *in vitro* expansion showed no predominating TCR Vβ usage for HLA-E_UL40_-specific T cells, indicative of an unbiased T cell response. A donor-specific focus revealed diverse and unique TCR Vβ chain repertoire in each host. Analysis of TCR Vα repertoire remains to be performed to fully define T-cell repertoire diversity.

*Ex vivo* phenotype analysis at distance from the infection revealed that HLA-E_UL40_ CD8 T cells belong to effector memory cells, most probably TEMRA, that display CD45RA^high^/CD45RO^low^. Chronic viral infections result in decreased function of virus-specific cellular and humoral immunity that occurs via upregulation of specific inhibitory receptors expressed on the immune cells. Our data showed that HLA-E_UL40_-restricted CD8 T cells express lower level of PD-1 as compared to HLA-A*02_pp65-_restricted CD8 T cells. It has been reported that CD8 T cells expressing high levels of co-inhibitory molecule PD-1 during the chronic infection are characterized by lower proliferation, cytokine production, and cytotoxic abilities [47]. PD-1 plays a significant role in establishment of virus-specific CD8 T-cell exhaustion and has been identified as a major regulator of T-cell exhaustion during chronic HIV/SIV infection [47]. Markedly upregulated on the surface of exhausted virus-specific CD8 T cells, PD-1 expression correlates with impaired virus-specific CD8 T-cell function and with elevated plasma viral load in chronic viral infections [48]. In our study, low levels of PD-1 expression compared to conventional HLA-A*02-restricted CD8 T cells appear as a feature of HLA-E-restricted CD8 T cells. The functional significance of the low PD-1 expression still requires investigations. It could be speculated that low PD-1 level on HLA-E_UL40_ CD8 T cells may reflect low TCR affinity as recently reported for antigen-specific CD8 T cells targeting melanoma peptides [49]. This feature could be important for homeostatic survival and proliferation to ensure long-term T cell survival [50].

It is interesting to notice that elected tropism of HCMV for endothelial cells also coincides with elevated basal level of HLA-E on this cell type as well as on hematopoietic cells as we previously reported [51]. Basal HLA-E expression can increase upon cellular stress caused by viral infection or heat shock and in inflammatory and cancer cells [41]. It can be speculated that HLA-E-expressing infected ECs play a role as both a trigger and a target of HLA-E-restricted CD8 T cells. We previously demonstrated *in vitro* the capacity of HLA-E_UL40_ CD8 T cells to efficiently kill primary allogeneic endothelial cell cultures presenting a homologous HLA signal peptide though HLA-E [30]. Consequently, HLA-E_UL40_ CD8 T cells could be involved in vascular injury and transplant rejection. The presence of UL40-specific CD8 T cells in the blood of lung transplant recipients was significantly associated with allograft dysfunction, which manifested as Bronchiolitis Obliterans Syndrome [31]. Although deciphering the clinical impact of HLA-E_UL40_ CD8 T cells was not the focus of the present study, clinical data indicated no significant impact on graft function (serum creatinine and proteinuria) at M12 post-transplantation (Table 1 and S1 Table). This could suggest that although we detected (by tetramer staining or TNF production) a multiplicity of HLA-E_UL40_ CD8 T-cell responses induced by HCMV and potentially cross-reactive toward a broad set of peptides including self and allogeneic HLA_sp,_ their activation may be either controlled by co-inhibitory receptors or functionally impaired. Another important finding in the setting of organ transplantation also emerges from our work. No HLA-E-restricted CD8 T cells were detected in HCMV^-^ transplant recipients suggesting that allograft does not induce *per se* consistent HLA-E-restricted CD8 T-cell response against allogeneic (i.e. donor) HLA-E_HLAsp_ complexes as speculated in earlier studies [19].

The function(s) of HLA-E_UL40_ CD8 T cells still remain to be established in regard to the control of HCMV infection. Efficient lysis of infected cells expressing high levels of HLA-E (i.e. endothelial cells, monocytes) could be a primary function expected for this effector CD8 T-cell subset. Regulatory functions for some HLA-E/Qa-1-restricted CD8 T-cell populations have been well established in mice [52] and more recently in humans [53]. Considering the high expression of HLA-E on CD4 T and B cells [54], a regulatory role for HLA-E_UL40_ CD8 T cells in the homoeostasis of anti-HCMV cellular immune response cannot be excluded beyond an action on the elimination of infected cells. Moreover, our findings provide evidence for self and allogeneic HLA peptides as potential targets and triggers (for their maintenance) of HLA-E_UL40_ CD8 T cells supporting effector and regulatory functions for these unconventional CD8 T cells beyond HCMV infection.

To conclude, HCMV UL40 induces specific HLA-E-restricted CD8 T-cell response with similar occurrence, magnitude, time course and long term persistence that pp65 viral protein. HLA-A*02 allele and *HLA-E* genotype are key determinants positively associated with HLA-E_UL40_ CD8 T cell response. HLA-E_UL40_ CD8 T cells are effector cells induced by HCMV in a strain-dependent manner that may specifically target and eliminate infected cells. We demonstrated that HLA-E_UL40_ CD8 T cells also display a potential reactivity toward self and allogeneic HLA peptides that may also contribute to the pathogenicity of HCMV, especially in immunocompromised patients.

## Materials and methods

### Ethics statement

Banked biological samples (PBMCs and DNAs) were issued from the DIVAT biocollection (CNIL agreement n°891735, French Health Minister Project n°02G55). This retrospective study was performed according to the guidelines of the local and national ethics committees (CCPRB, CHU de Nantes, France). Blood samples collected from anonymous healthy volunteers (n = 25) were obtained from the Etablissement Français du Sang (EFS Pays de la Loire, Nantes) and collected with donor’s specific and written informed consent for research use.

### Clinical and demographic characteristics of the cohort

A total of 121 patients who underwent kidney (105/121) or kidney-pancreas (16/121) transplantation in our center (Institut de Transplantation/Urologie/Nephrologie, ITUN, CHU de Nantes, France) between 2006 and 2013 were retrospectively enrolled in our cohort study. The cohort includes 4 groups of transplant recipients defined by their HCMV status (**Table 1**). The groups were defined according to the HCMV serology of the recipient (HCMV^-^ or HCMV^+^) and for HCMV^+^ the status of infection (primary, latent, reactivation) at M12 post-transplantation: HCMV active infection (AI) was defined by having at least two consecutive PCR with a viral load (VL) > 3 log10, expressed as number of viral DNA copies (log10cop) per 10^6^ cells. No statistical difference (p > 0.05) between the groups was found related to the age of the recipients at the day of transplantation, gender ratio, frequency of HLA-A*02 genotype, and the post-transplant time for the blood samples. There is also no statistical difference between the groups concerning the gender ratio of transplant donors, the rank of the transplantation and the duration of cold ischemia. Mismatches of total HLA-I and/or HLA-II for each donor/recipient pairs were equal in the groups. Finally, expected statistical differences between the groups only appeared related to HCMV primary infection status at 12 months post-transplantation. Healthy HCMV^+^ seropositive blood donors (n = 25) were also recruited in this study. No statistical differences were founded between HV and KTR patients related to age or gender ratio.

### Blood samples and PBMC isolation

Frozen PBMCs isolated from blood samples issued from kidney transplant recipients were prospectively stored at the Centre de Ressources Biologiques (CRB, Nantes, France). Cells were thawed 24 hours before use in RPMI-1640 medium (Gibco, Saint Aubin, France) supplemented with 8% human serum, 2 mM L-glutamine (Gibco), 100 U/mL penicillin (Gibco), 0.1 mg/mL streptomycin (Gibco) and 50 U/mL human recombinant IL-2 (Proleukin, Novartis Pharma, Rueil-Malmaison, France). Blood samples from HCMV^+^ HV’s were provided by the Clinical Development and Transfer Facility (DTC Facility, INSERM/SFR Federative Structure Research Francois Bonamy, Nantes, France). PBMC were isolated by Ficoll density gradient (Eurobio, Les Ulis, France) and used immediately.

### HCMV monitoring and HCMV UL40 sequencing

For HCMV monitoring, EDTA blood samples were collected for blood donation from healthy volunteers, patient’s follow up or during the acute phase of HCMV infection. HCMV serology was performed using the LIAISON CMV IgG; LIAISON CMV IgM and LIAISON CMV IgG Avidity tests (Diasorin, Saluggia, Italy). Additional evidence of active HCMV replication was examined using an in-house real time HCMV PCR in whole blood, adapted from [55]. The combination of positive CMV IgG, positive IgM, and positive PCR was used for confirmation of primary HCMV infection. For UL40 sequencing, DNA were extracted using QIAsymphony system (Qiagen, Courtaboeuf, France) from 200μL of whole blood samples with the QIAamp DSP DNA Mini Kit (Qiagen). The HCMV UL40 region (858bp) was amplified by PCR using a protocol adapted from [56]. Briefly, the following specific forward and reverse primers were used for a long PCR: forward 5’-TCCTCCCTGGTACCCGATAACAG-3’ and reverse 5’-CGGGCCAGGACTTTTTAATGGCC-3’. Standard reaction mixtures were realized using SYBRPremix Ex Taq kit (Takara Bio Europe, Saint-Germain-en-Laye, France), with the following PCR parameters: one cycle of 94°C for 12 min, then 50 cycles of 94°C 30 sec, 63°C 30 sec and 72°C for 1 min 30, and finally one cycle of 72°C 7 min. PCR products were analyzed by electrophoresis through a 9% non-denaturing acrylamide-bisacrylamide 37.5–1 gel stained with ethidium bromide. PCR products were purified using the enzymatic method ExoSAP-IT USB (Affymetrix, Thermo Fisher Scientific, Villebon-sur-Yvette, France). Bidirectional sequence was performed using the fluorescent BigDye terminator method (Big Dye version 1.1 Cycle Sequencing Kit, Applied Biosystems, Courtaboeuf, France) and sequencing reactions were run on Applied Biosystems ABI Prism 3130 XL. Nucleotide and amino acid sequences analyses were performed using Seqscape software (version 2.5, Applied Biosystems). All sequences were imported and aligned in MEGA5 software using the UL40 sequence from Human Herpesvirus 5 (Merlin strain) as reference sequence (NCBI Reference Sequence: NC_006273.2). Sequence LOGO were created using the Los Alamos HIV Database tool Analyse Align (http://www.hiv.lanl.gov/content/sequence/ANALYZEALIGN/analyze_align.html), which was based on WebLOGO3.

### *HLA-E* and *HLA-A*, *-B*, *-C* genotyping

Banked genomic DNAs (gDNAs) from the transplant donor/recipient pairs (n = 121) analysed in this study and available in the DIVAT biocollection (62 donors and 106 recipients) were harvested. Genomic DNA was extracted from blood samples issued from the HCMV^+^ HV (n = 25) using usual proteinase K/phenol-chloroform method and subsequently used for genotyping. For HLA-E*01:01 and HLA-E*01:03 determination, a first PCR product was generated from gDNA encompassing exon1 to 3 coding for the alpha domains and using the following primers: forward 5'-TCCTGGATACTCATGACGCAGACTC-3’ and reverse 5'-CCTCTTACCCAGGTGAAGCAGCG-3’. Next, a second run of amplification was performed into two separated nested PCR targeting exons 1–2 and exon 3, respectively with the primer pairs: 5-'TCCTGGATACTCATGACGCAGACTC-3’ and 5'-ATCTGGGACCCGAAGATTCGA-3’, 5'-TCGAATCTTCGGGTCCCAGAT-3’ and 5'-CCTCTTACCCAGGTGAAGCAGCG-3’. DNA sequencing was performed with BigDye Terminator v3.1 kit (Applied Biosystems) according to the manufacturer's instructions on the DNA Sequencing Core Facility (INSERM/SFR François Bonamy, Nantes, France), using a 48-capillary Applied Biosystems 3730 automatic system (Applied Biosystems). Sequences were analyzed using Chromas 2.33 software (Digital River GmbH, Shannon, Ireland) using a SNP at AA position 107 to discriminate between *01:01 and *01:03 alleles. HLA-A,-B,-C genotypes of transplant donors/recipients pairs and HV were performed by either the EFS (Nantes, Pays de la Loire) or Histogenetics (Ossining, NY, USA), by using PCR-SSO (and completed by PCR-SSP if necessary) and based on the IMGT/HLA database nomenclature (www.ebi.ac.uk/ipd/imgt/hla/).

### Production of HLA-E/UL40_15-23_ and HLA-A*02:01/pp65_495-503_ tetramer complexes

Nine-mers UL40_15-23_ peptides from 11 different HCMV strains (VMAPRTLIL, VMAPRTLLL, VMAPRTLVL, VMAPRTVLL, VMAPRSLIL, VMAPRSLLL, VMTPRTLVL, VMAPQSLLL, VTAPRTLLL, VTAPRTVLL, VMAPRALLL) and the UL83 pp65_495-503_ peptide (NLVPMVATV) were synthesized (purity>95%) and purchased from Proteogenix SAS (Schiltigheim, France). HLA-E*01:01/UL40_15-23_ (HLA-E_UL40_) and HLA-A*02:01/pp65_495-503_ (HLA-A*02_pp65_) complexes were generated as described previously [57]. Recombinant HLA proteins were produced in *E*.*coli* and refolded with 15μg/mL of each UL40_15-23_ peptide for HLA-E-monomers or pp65_495-503_ peptide for HLA-A*02-monomers. Next, HLA-monomers were biotinylated for 4h at 30°C with 6μg/mL BirA (Immunotech, Marseille, France), purified and tetramerized with BV421- or APC-labelled streptavidin (BD Biosciences, Le Pont de Claix, France). Tetramerization was confirmed by gel filtration chromatography (Superdex 200 column, Sigma-Aldrich, Saint-Quentin Fallavier, France).

### *Ex vivo* detection and quantification of HLA-E_UL40_ and HLA-A*02_pp65_ T cells by flow cytometry

To investigate the frequency of the anti-HCMV CD8 T-cell responses in individuals, PBMC (3x10^5^ per condition) were pre-incubated with a blocking anti-CD94 mAb (clone HP-3D9, 5 μg/mL, BD Biosciences) for 20 min at 4°C to completely abrogate the non-specific staining of CD94/NKG2^+^ T cells by HLA-E-tetramers (**S1 Fig**). PBMCs were then incubated with one of the different BV421-labelled HLA-E- or HLA-A*02-tetramers (10 μg/mL, 30min, 4°C), before costaining (30min, 4°C) with the following antibodies: anti-CD3 (clone SK7/Leu4, BV786, 2 μg/mL, BD Biosciences), anti-CD8α (clone RPA-T8, BV650, 0.1 μg/mL, BD Biosciences) and anti-TCR γδ (clone 11F2, APC-Vio770, 3 μg/mL, Miltenyi Biotec, Paris, France). Dead cells were excluded using NucRed Dead 647 ready probes reagent (Life technologies). As a control of tetramer staining, a FMO condition (Fluorescence Minus One; all labelled-markers except one) without tetramers was performed for each sample. Acquisition was performed on a BD LSR II and analyses were performed using BD DIVA Software v6.0 as described below. Compensations were performed by using anti-mouse κ chain Ab-coated beads (anti-mouse Ig, κ chain/negative control compensation particles set, BD Biosciences) incubated with corresponding Ab at the same concentration during 15 min at room temperature. Data acquisition for the 121 KTR and 25 HV was normalized with application settings based on the KTR#001 patient. Gating analysis strategy was identical for all samples (S1 Fig).

### *Ex vivo* analysis of time course and activation of anti-HCMV CD8 T-cell responses post-infection

To follow-up the development of HCMV-specific T-cell subpopulations in KTR, banked PBMCs from 16 KTR prospectively collected at 1, 2, 3, 4, 5, 6, 7, 9, 10,12 and 13 months post-transplantation were used. For each time point tested, UL40-specific HLA-E-restricted (HLA-E_UL40_) and pp65-specific HLA-A*02:01-restricted (HLA-A*02_pp65_) T cells were concomitantly stained and quantified as described above with the complementary mAbs: anti-CD69 (clone FN50, BUV395, 2 μg/mL, BD Biosciences) and anti-PD1 (clone EH12 (.1), PE, 2 μg/mL, BD Biosciences). Acquisition and analysis was performed on a BD LSR Fortessa X-20 with BD DIVA Software v8.0. Longitudinal samples for each patient were all stained and acquired in the same experiment.

### *In vitro* expansion of UL40-specific HLA-E-restricted T cells

HLA-E_UL40_ T cells were sorted for 5 transplant recipients (#104, #105, #107, #108 and #109) from PBMCs harvested at 12 months post-transplantation as previously described [58]. Briefly, streptavidin-coated beads (Dynabeads M-280 Streptavidin, Invitrogen, Villebon sur Yvette, France) were saturated with HLA-E/UL40_15-23_ monomers before incubation with PBMCs (5x10^6^) for 4h. The UL40_15-23_ peptide corresponding to the own HCMV infecting strain was selected for each patient. HLA-E_UL40_ T cells were isolated by immunomagnetic sorting and then expanded for 21–30 days as follows: cells were seeded in 96-well plates (3x10^3^/well) and stimulated with phytohemagglutinin (1 μg/mL, PHA-L; Sigma-Aldrich) in the presence of irradiated EBV-transformed B-cell lines and allogeneic PBMC from healthy donors (EFS, Nantes) as feeder. Cells were grown in RMPI-1640 medium supplemented with 8% human serum, 2 mM L-glutamine, 100 U/mL penicillin and 0.1 mg/mL streptomycin and human recombinant IL-2 (150 U/mL). Purity (>95%) of each T cell population was defined after 14 days of culture by tetramer staining.

### *In vitro* functional analysis of UL40-specific HLA-E-restricted T-cell activity and peptide-specificity

The use of tetramers to activate T cells has been extensively reviewed by Wooldridge and colleagues [59]. T-cell activation by soluble peptide–MHC-I tetramers is very sensitive for inducing a full range of effector functions. In addition to inducing a normal pattern of T-cell signaling [60] tetramer activation results in lytic granule release, a full profile of cytokine and chemokine release and the production of a wide range of cell surface activation markers [61]. In the present study, a series of preliminary experiments were performed to set up the assay measuring T-cell activation in response to HLA-E/UL40 peptide tetramers. Representative results from these preliminary assays are illustrated in the S4 Fig. To determine the peptide specificity of HLA-E_UL40_-restricted T cells, purified cell lines (1x10^5^ cells /condition) were stimulated for 5h at 37°C in 96-wells plates with one of the 11 HLA-E/UL40-tetramers at 20 μg/mL in RPMI 1640 medium in the presence of Brefeldin A (10 μg/mL, Sigma). Next, cells were incubated with an anti-CD8α mAb (clone RPA-T8, 1 μg/mL, BioLegend) for 30 min at 4°C before fixation with 4% paraformaldehyde. After permeabilization with 0.1% (w/v) saponin (Sigma-Aldrich), cells were stained for 30 min at room temperature with an anti-TNFα mAb (clone cA2, Miltenyi). Cells were finally washed twice in PBS-0.1% (v/v) BSA and 0.1% (w/v) saponin before sample acquisition on BD FACS Canto II.

### Phenotype analysis of UL40-specific HLA-E-restricted T cells

Phenotypic analyses were performed on PBMCs from 3 patients. Analysis of T cells before activation was performed *ex vivo* using the following mAbs: anti-CD3 (clone UCHT1), anti-TCR αβ (clone T10B9.1A-31/T10B9), anti-TCR γδ (clone B1), anti-CD45RA (clone HI100), anti-CD45RO (clone UCHL1), anti-CD28 (clone CD28.2), anti-CD27 (clone M-T271), anti-CD57 (clone NK-1) from BD Biosciences; anti-CD8β (clone SIDI8BEE) from eBioscience (Thermo-Fisher); anti-CD4 (clone RPA-T4), anti-CD8α (clone RPA-T8) from Miltenyi and anti-CCR7 (clone 150503) from R&D Systems. For Vβ TCR repertoire analysis, purified HLA-E_UL40_ T cells (2x10^5^) were incubated 30 min at 4°C in PBS-0.1% (v/v) BSA with the TCR Vβ Repertoire Kit (IO Test Beta Mark–TCR Vβ Repertoire Kit, Beckman Coulter, Villepinte, France). This kit allows detection of the following Vβ TCR: 1, 2, 3, 4, 5.1, 5.2, 5.3, 7.1, 7.2, 8, 9, 11, 12, 13.1, 13.2, 13.6, 14, 16, 17, 18, 20, 21.3, 22 and 23. All Abs were used at saturating concentration conforming to the manufacturer’s recommendation.

### Statistical analyses

Data are expressed as medians + interquartile range between Q1 and Q3, or percentages. Appropriate non-parametric statistical analysis (Kruskall-Wallis test, Mann-Whitney, Fischer’s exact test or Pearson’s chi-squared test with adequate post-tests) was performed using GraphPad Prism(GraphPad, San Diego, CA) and R softwares. The type I error rate *α* (probability threshold of rejecting the null hypothesis given that it is true) was set to 0.05. A *p-*value *<*0.05 was considered to represent a statistically significant difference.

## Supporting information

S1 Table Kidney graft function in HCMV^+^ recipients with or without HLA-E_UL40_ CD8 T cell responses. (PDF)

S1 Fig HLA-E_UL40_ T-cell detection assay. (A-B-C) CD94 blockade using an anti-CD94 monoclonal antibody to avoid HLA-E tetramer binding to CD94/NKG2A and CD94/NKG2C receptors. (A) CD94/NKG2A (left, upper panel) and CD94/NKG2C (left, lower panel) expression on lymphocyte-gated PBMCs from an HCMV^+^ HV (representative data from a single donor are shown) that display both CD94/NKG2A^+^ and high CD94/NKG2C^+^ NK responses (left panel) but no HLA-E-restricted T-cell response. (B) HLA-E_VMAPRTLIL_ tetramer staining was performed either in the absence (left panel) or in the presence (right panel) of anti-CD94 monoclonal antibody on PBMCs from the same HCMV^+^ HV. CD3^-^ cells–including NK cells–were represented on top panel and T cells (CD3^+^ cells) on bottom panel. These data show that incubation with blocking anti-CD94 mAb totally abrogates binding of tetramer to CD3^-^ and CD3^+^ T cells. Since we used a fluorochrome-labeled blocking anti-CD94, in the absence (-) of antibody, CD94 is not detected. In this sample no HLA-E_UL40_ T cells were detected. Similar results were obtained for each of the eleven different HLA-E_UL40_ tetramers used in this study and confirmed with PBMCs from two other HV. (C) Detection of HLA-E_UL40_-specific CD8 T cells after blocking CD94. Representative examples of HLA-E/peptide tetramer staining before and after blocking CD94, with specific anti-CD94 mAb, on PBMCs either without (HCMV^-^ healthy volunteer, upper panel) or with a HLA-E_UL40_ CD8 T-cell response (HCMV^+^ individuals, lower panel) are shown. HLA-E/peptide tetramer staining was analyzed after gating on CD3^-^ cells, to investigate NK cells, on γδ T cells and on αβ CD8^+^ T cells. In PBMC from HCMV^-^ donor, HLA-E/peptide tetramers bind to a fraction of CD3^-^ and γδ T cells through interaction with CD94/NKG2A or CD94/NKG2C receptors usually expressed on these subsets. HLA-E/peptide tetramers staining on CD3^-^ and γδ T cells was abrogated after blocking CD94 with anti-CD94 mAb. Similar inhibition of HLA-E/peptide tetramers staining on CD3^-^ cells and γδ T cells was observed for HCMV^+^ hosts. However, blockade of CD94 preserves the specific binding of HLA-E/peptide tetramers to αβ TCR and thus allows the detection of HLA-E_UL40_ CD8 T cells. (D) Sensitivity of the detection assay. Two monoclonal T-cell populations were used: one specific of the HLA-E/VMAPRTLIL (UL40_15-23_ viral peptide) and the other specific of the HLA-A*02/NLVPMVATV (pp65 _495–503_ viral peptide). These T cells were diluted in PBMCs from healthy donor at different ratios (0, 0.1, 1 and 10%). For detection assay, cells were preincubated with a blocking anti-CD94 mAb before co-staining with the relevant tetramer/peptide in combination with anti-CD3, -TCRγδ; and -CD8 mAbs. Tetramers^+^ CD8 cells were gated on live CD3^+^TCRγδ^-^ cells. (E) Gating strategy for the *ex vivo* analysis of HLA-E_UL40_- or HLA-A*02_pp65_-specific CD8 T cells on PBMCs. Lymphocytes were gated on the basis of their morphology in FSC-A/SSC-A (1), and doublets of cells were excluded using FSC-A/FSC-H (2) and SSC-A/SSC-H (3) dot plots. Dead cells were excluded (4) and after gating on the CD3^+^ TCR γδ^-^ cells (5), frequency of tetramers^+^ CD8α^+^ T-cell subpopulations was determined (6).
(PDF)

S2 Fig Analysis of HCMV UL40 sequence polymorphism in HCMV^+^ kidney transplant recipients. Genomic DNAs isolated from HCMV positive blood samples of HCMV^+^ transplant recipients (n = 25) were sequenced for the identification of UL40 protein (amino acids 1–221) provided by the circulating HCMV strains. Amino acid variability, expressed as a number of amino acid variants (A) and in percentages (B), within the HLA-E-binding peptide (UL40_15-23_, shown in red) among the sequence for HCMV UL40 signal peptide (UL40_1-37_, shown in grey) and the coding sequence (UL40_37-221_, shown in black). A total of 32 UL40 sequences from 25 hosts were analysed. UL40 protein sequence from the Merlin HCMV strain (NCBI Reference Sequence: NC_006273.2) was used as reference.
(PDF)

S3 Fig Longitudinal analysis of PD-1 and CD69. Expression of PD-1 (A) and CD69 (B) analysed on blood samples from KTR#008, #107 and #108 at different time points post-transplantation. Data represent the % of CD69^+^/tetramer^+^ and PD-1^+^/tetramer^+^ cells among CD3^+^ CD8α^+^ TCRγδ^-^ tetramers^+^ cells, for HLA-E_UL40_ (in blue) and HLA-A*02_pp65_ (in red) anti-HCMV CD8 T-cell responses post-transplantation.
(PDF)

S4 Fig Representative phenotypes of HLA-E_UL40_ CD8 T cells. Immunostaining for CD45RO, CD45RA, CD27, CD28, CCR7 and CD57 were performed ex vivo on PBMCs by co-staining with HLA-E_UL40_ tetramers and after gating on tetramer^+^ CD3^+^, γδ^-^ T, CD8^+^ T cells. HLA-E_UL40_ CD8 T cells detected in PBMCs, harvested at M12 post-graft, from 2 HCMV^+^ kidney transplant recipients (KTR #107 and KTR#109) and representative from 3 KTRs are shown.
(PDF)

S5 Fig Antigen-specific CD8 T-cell activation using HLA-E/peptide tetramer complexes. TNF production (% of positive cells) detected by intracellular staining on CD8 T cells gated from PBMCs either unstimulated or stimulated with soluble HLA-E monomers, HLA-E monomers coated on M280 Dynabeads or HLA-E tetramers for 5h. HLA-E molecules were loaded with either an irrelevant peptide (upper panel) or with the specific peptide target (lower panel). This figure shows that an irrelevant peptide gives no response indicating the specificity of the method. When the ability of tetramers *versus* monomers loaded with specific peptide target to stimulate HLA-E_UL40_ CD8 T cells was compared we found a higher percentage of TNF-producing CD8 T cells with HLA-E tetramer/peptides (52.4%) compared to HLA-E monomer/peptides (22.6% and 10.2% for uncoated and bead-coated, respectively).
(PDF)
