# Supplementary material for: HCMV triggers frequent and persistent UL40-specific unconventional HLA-E-restricted CD8 T-cell responses with potential autologous and allogeneic peptide recognition
Source: PLoS Pathog. 2018 Apr 30;14(4):e1007041. doi: 10.1371/journal.ppat.1007041 (PMC5945056; doi:10.1371/journal.ppat.1007041)
Supplement: S1 Table — (PDF) [file ppat.1007041.s001.pdf]

S1 Table

	HLA-E$_{UL40}$ CD8 T-cell response		
	- (n=57)	+ (n=23)	p-value
Serum Creatinine at M12 [µmol/L; median (Q1-Q3)]	137 (118-175)	134 (96-166)	0.3371[1]
Proteinuria at M12 [g/24h; median (Q1-Q3)]	0.23 (0.11-0.46)	0.28 (0.12-0.44)	0.7133[1]

[1] Mann Whitney test
