# Supplementary figures and images for: HCMV triggers frequent and persistent UL40-specific unconventional HLA-E-restricted CD8 T-cell responses with potential autologous and allogeneic peptide recognition
Source: PLoS Pathog. 2018 Apr 30;14(4):e1007041. doi: 10.1371/journal.ppat.1007041 (PMC5945056; doi:10.1371/journal.ppat.1007041)

**S1 Fig**

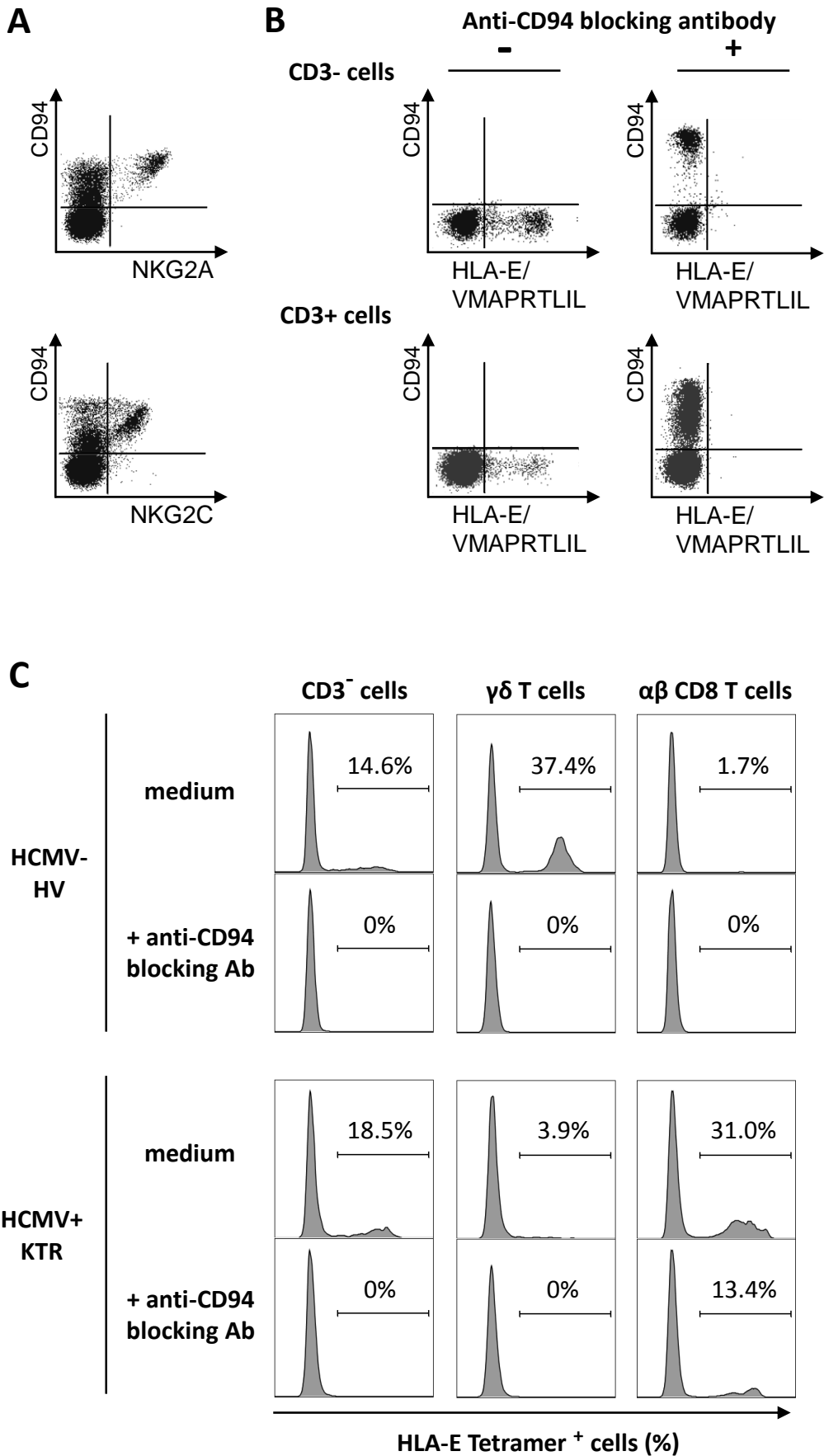

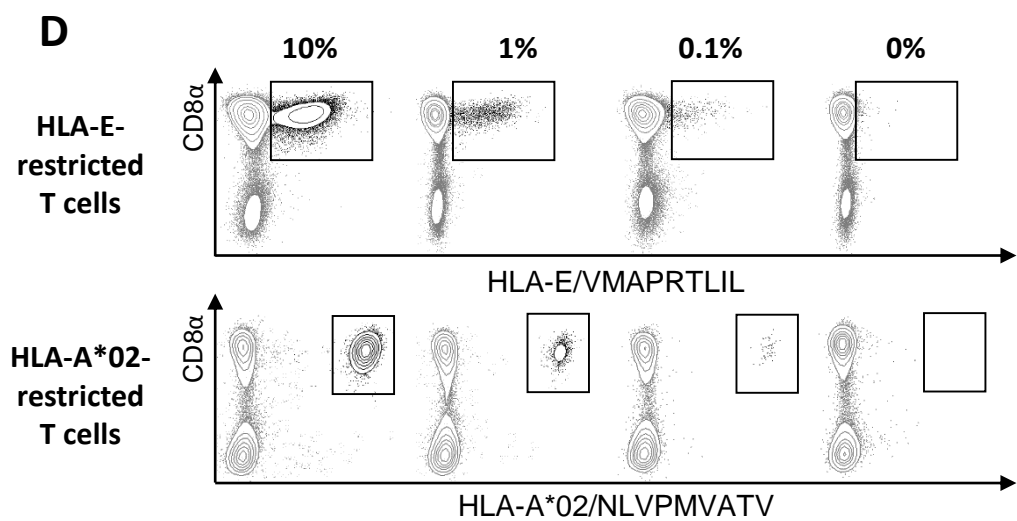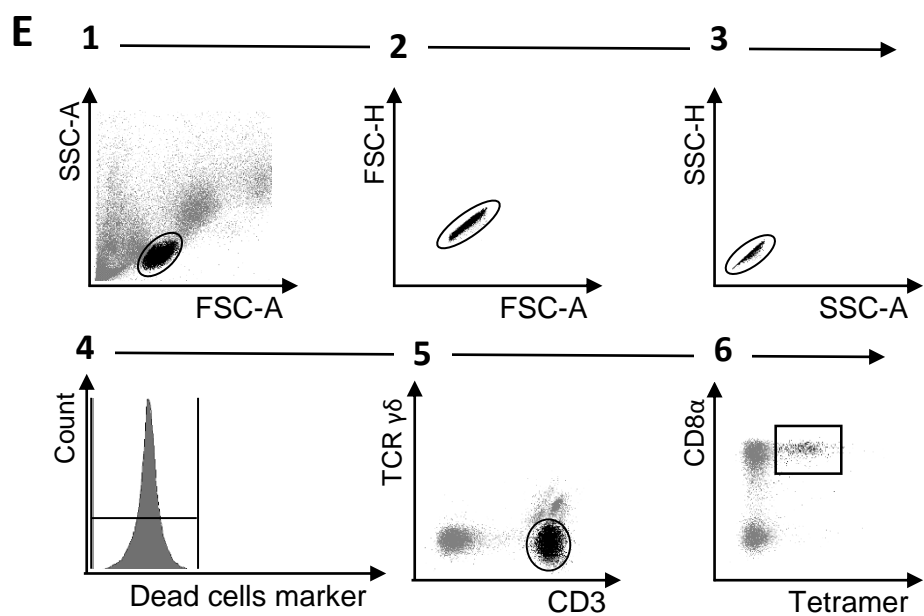

Supplement: S1 Fig — (A-B-C) CD94 blockade using an anti-CD94 monoclonal antibody to avoid HLA-E tetramer binding to CD94/NKG2A and CD94/NKG2C receptors. (A) CD94/NKG2A (left, upper panel) and CD94/NKG2C (left, lower panel) expression on lymphocyte-gated PBMCs from an HCMV+ HV (representative data from a single donor are shown) that display both CD94/NKG2A+ and high CD94/NKG2C+ NK responses (left panel) but no HLA-E-restricted T-cell response. (B) HLA-EVMAPRTLIL tetramer staining was performed either in the absence (left panel) or in the presence (right panel) of anti-CD94 monoclonal antibody on PBMCs from the same HCMV+ HV. CD3- cells–including NK cells–were represented on top panel and T cells (CD3+ cells) on bottom panel. These data show that incubation with blocking anti-CD94 mAb totally abrogates binding of tetramer to CD3- and CD3+ T cells. Since we used a fluorochrome-labeled blocking anti-CD94, in the absence (-) of antibody, CD94 is not detected. In this sample no HLA-EUL40 T cells were detected. Similar results were obtained for each of the eleven different HLA-EUL40 tetramers used in this study and confirmed with PBMCs from two other HV. (C) Detection of HLA-EUL40-specific CD8 T cells after blocking CD94. Representative examples of HLA-E/peptide tetramer staining before and after blocking CD94, with specific anti-CD94 mAb, on PBMCs either without (HCMV- healthy volunteer, upper panel) or with a HLA-EUL40 CD8 T-cell response (HCMV+ individuals, lower panel) are shown. HLA-E/peptide tetramer staining was analyzed after gating on CD3- cells, to investigate NK cells, on γδ T cells and on αβ CD8+ T cells. In PBMC from HCMV- donor, HLA-E/peptide tetramers bind to a fraction of CD3- and γδ T cells through interaction with CD94/NKG2A or CD94/NKG2C receptors usually expressed on these subsets. HLA-E/peptide tetramers staining on CD3- and γδ T cells was abrogated after blocking CD94 with anti-CD94 mAb. Similar inhibition of HLA-E/peptide tetramers staining on CD3- cells and γδ T cells [file ppat.1007041.s002.pdf]

**S2 Fig**

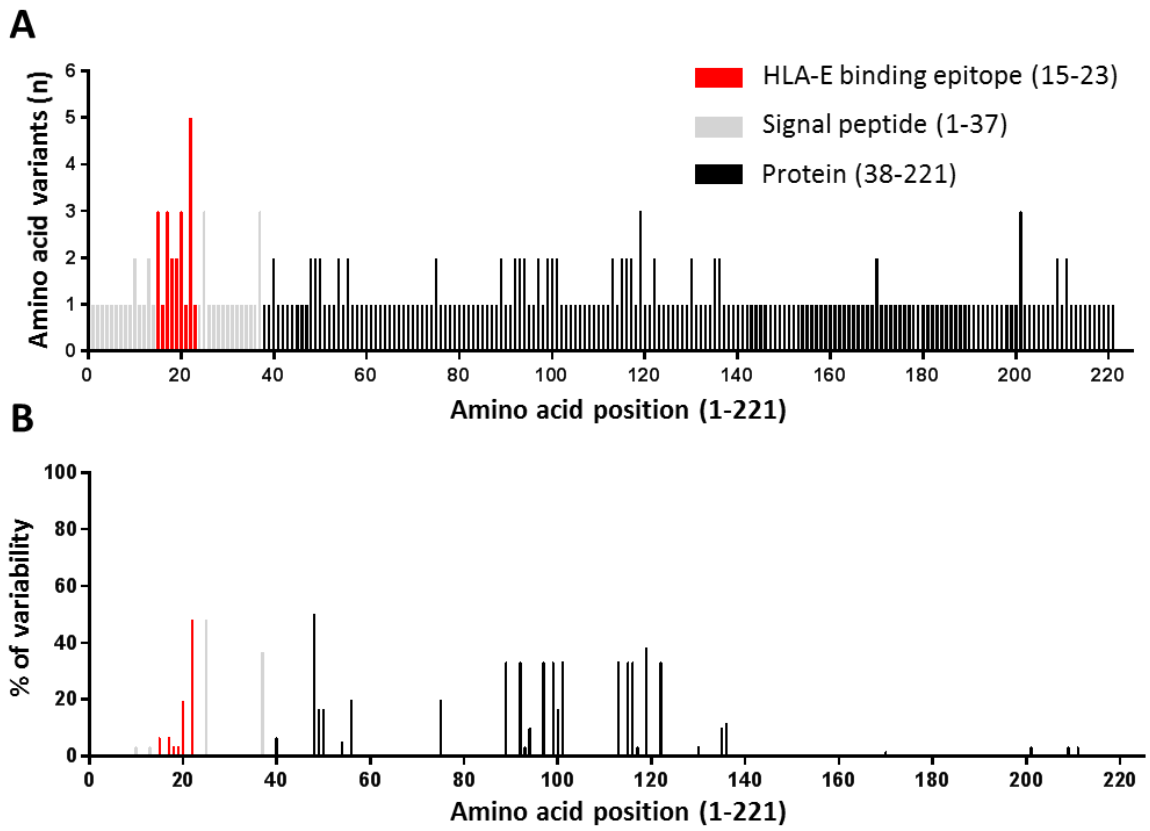

Supplement: S2 Fig — Genomic DNAs isolated from HCMV positive blood samples of HCMV+ transplant recipients (n = 25) were sequenced for the identification of UL40 protein (amino acids 1–221) provided by the circulating HCMV strains. Amino acid variability, expressed as a number of amino acid variants (A) and in percentages (B), within the HLA-E-binding peptide (UL4015-23, shown in red) among the sequence for HCMV UL40 signal peptide (UL401-37, shown in grey) and the coding sequence (UL4037-221, shown in black). A total of 32 UL40 sequences from 25 hosts were analysed. UL40 protein sequence from the Merlin HCMV strain (NCBI Reference Sequence: NC_006273.2) was used as reference. (PDF) [file ppat.1007041.s003.pdf]

S3 Fig

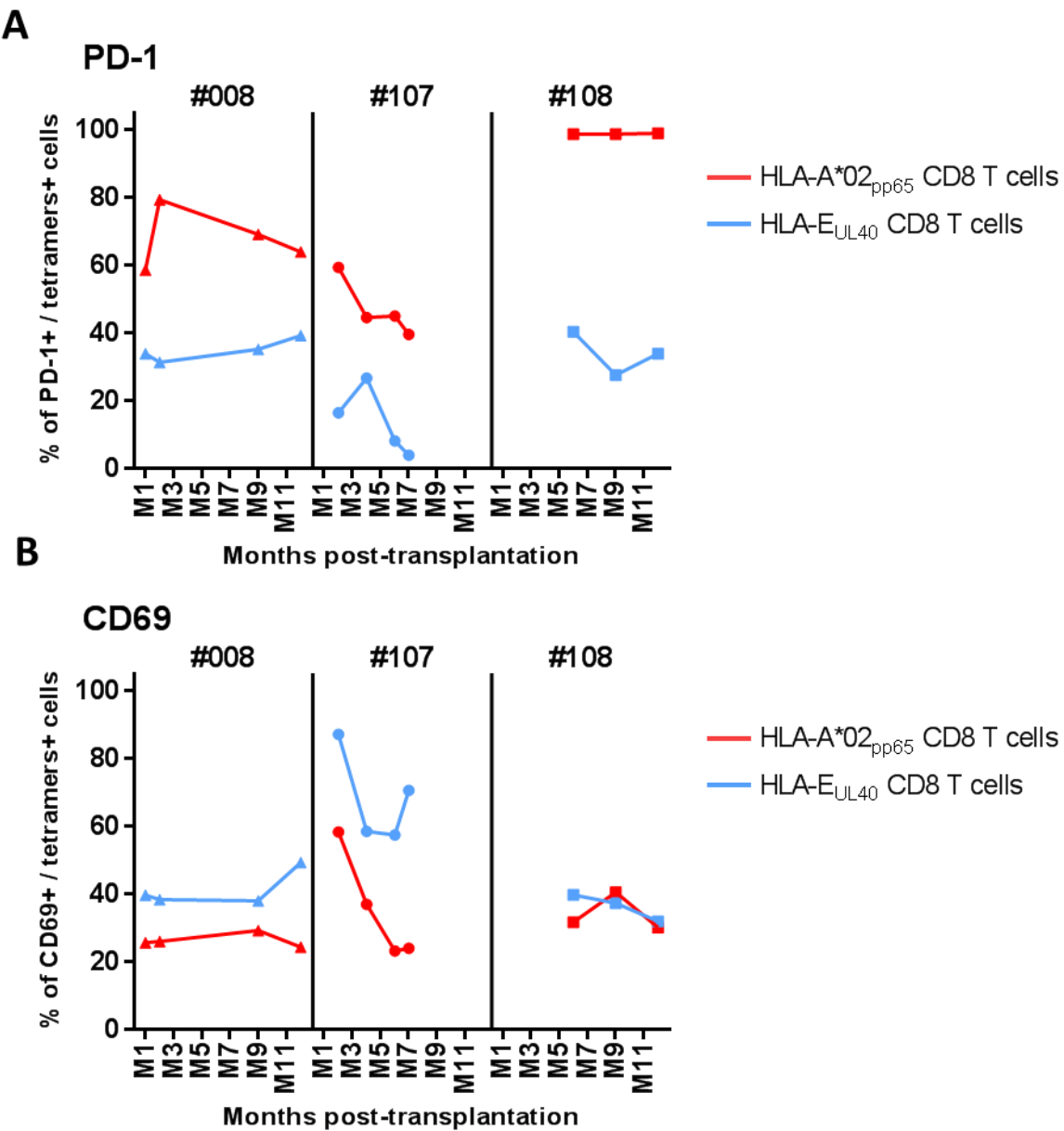

Supplement: S3 Fig — Expression of PD-1 (A) and CD69 (B) analysed on blood samples from KTR#008, #107 and #108 at different time points post-transplantation. Data represent the % of CD69+/tetramer+ and PD-1+/tetramer+ cells among CD3+ CD8α+ TCRγδ- tetramers+ cells, for HLA-EUL40 (in blue) and HLA-A*02pp65 (in red) anti-HCMV CD8 T-cell responses post-transplantation. (PDF) [file ppat.1007041.s004.pdf]

S4 Fig

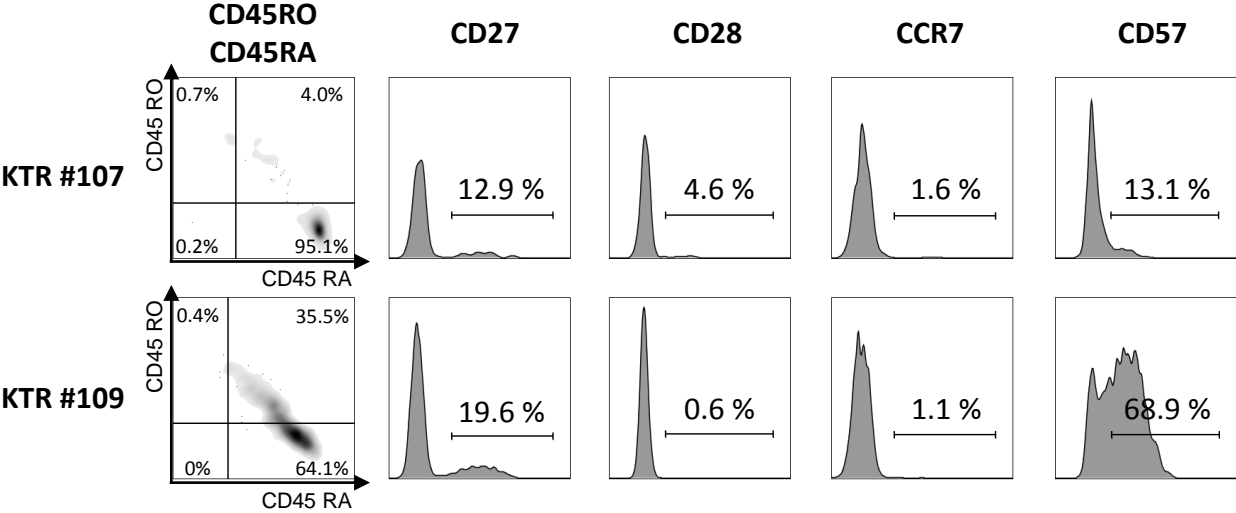

Supplement: S4 Fig — Immunostaining for CD45RO, CD45RA, CD27, CD28, CCR7 and CD57 were performed ex vivo on PBMCs by co-staining with HLA-EUL40 tetramers and after gating on tetramer+ CD3+, γδ- T, CD8+ T cells. HLA-EUL40 CD8 T cells detected in PBMCs, harvested at M12 post-graft, from 2 HCMV+ kidney transplant recipients (KTR #107 and KTR#109) and representative from 3 KTRs are shown. (PDF) [file ppat.1007041.s005.pdf]

S5 Fig

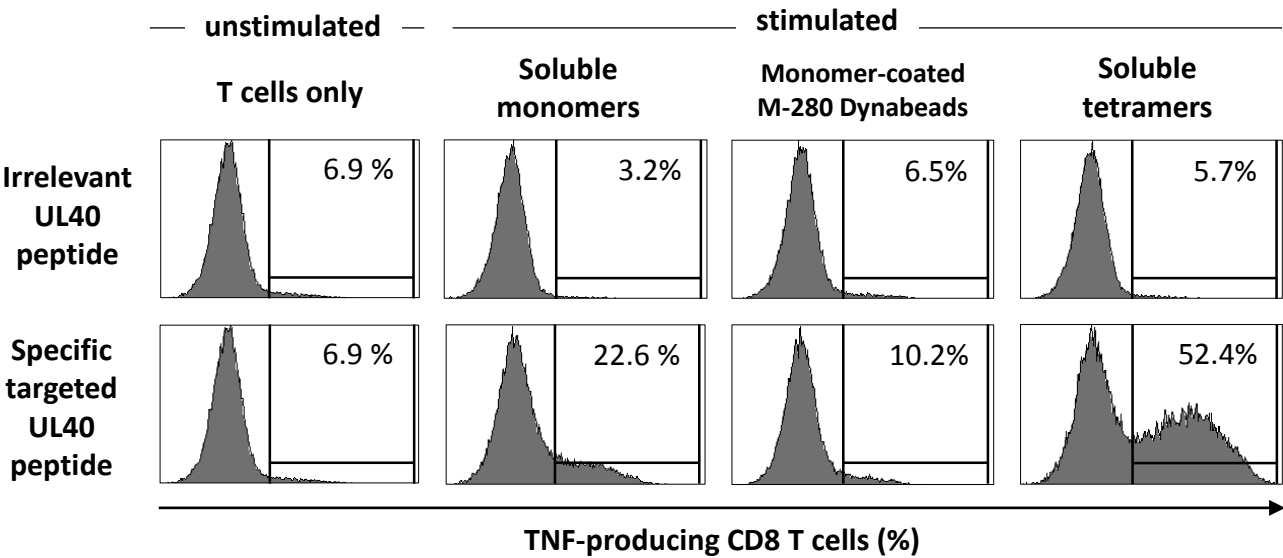

Supplement: S5 Fig — TNF production (% of positive cells) detected by intracellular staining on CD8 T cells gated from PBMCs either unstimulated or stimulated with soluble HLA-E monomers, HLA-E monomers coated on M280 Dynabeads or HLA-E tetramers for 5h. HLA-E molecules were loaded with either an irrelevant peptide (upper panel) or with the specific peptide target (lower panel). This figure shows that an irrelevant peptide gives no response indicating the specificity of the method. When the ability of tetramers versus monomers loaded with specific peptide target to stimulate HLA-EUL40 CD8 T cells was compared we found a higher percentage of TNF-producing CD8 T cells with HLA-E tetramer/peptides (52.4%) compared to HLA-E monomer/peptides (22.6% and 10.2% for uncoated and bead-coated, respectively). (PDF) [file ppat.1007041.s006.pdf]
